# Constraints on anomalous Higgs boson couplings from its production and decay using the WW channel in proton–proton collisions at $\sqrt{s} = 13~\text {TeV}$

**DOI:** 10.1140/epjc/s10052-024-12925-0

**Published:** 2024-08-05

**Authors:** CMS Collaboration, A. Hayrapetyan, A. Tumasyan, W. Adam, J. W. Andrejkovic, T. Bergauer, S. Chatterjee, K. Damanakis, M. Dragicevic, P. S. Hussain, M. Jeitler, N. Krammer, A. Li, D. Liko, I. Mikulec, J. Schieck, R. Schöfbeck, D. Schwarz, M. Sonawane, S. Templ, W. Waltenberger, C.-E. Wulz, M. R. Darwish, T. Janssen, P. Van Mechelen, E. S. Bols, J. D’Hondt, S. Dansana, A. De Moor, M. Delcourt, H. El Faham, S. Lowette, I. Makarenko, D. Müller, A. R. Sahasransu, S. Tavernier, M. Tytgat, G. P. Van Onsem, S. Van Putte, D. Vannerom, B. Clerbaux, A. K. Das, G. De Lentdecker, L. Favart, P. Gianneios, D. Hohov, J. Jaramillo, A. Khalilzadeh, K. Lee, M. Mahdavikhorrami, A. Malara, S. Paredes, L. Pétré, N. Postiau, L. Thomas, M. Vanden Bemden, C. Vander Velde, P. Vanlaer, M. De Coen, D. Dobur, Y. Hong, J. Knolle, L. Lambrecht, G. Mestdach, K. Mota Amarilo, C. Rendón, A. Samalan, K. Skovpen, N. Van Den Bossche, J. van der Linden, L. Wezenbeek, A. Benecke, A. Bethani, G. Bruno, C. Caputo, C. Delaere, I. S. Donertas, A. Giammanco, K. Jaffel, Sa. Jain, V. Lemaitre, J. Lidrych, P. Mastrapasqua, K. Mondal, T. T. Tran, S. Wertz, G. A. Alves, E. Coelho, C. Hensel, T. Menezes De Oliveira, A. Moraes, P. Rebello Teles, M. Soeiro, W. L. Aldá Júnior, M. Alves Gallo Pereira, M. Barroso Ferreira Filho, H. Brandao Malbouisson, W. Carvalho, J. Chinellato, E. M. Da Costa, G. G. Da Silveira, D. De Jesus Damiao, S. Fonseca De Souza, R. Gomes De Souza, J. Martins, C. Mora Herrera, L. Mundim, H. Nogima, J. P. Pinheiro, A. Santoro, A. Sznajder, M. Thiel, A. Vilela Pereira, C. A. Bernardes, L. Calligaris, T. R. Fernandez Perez Tomei, E. M. Gregores, P. G. Mercadante, S. F. Novaes, B. Orzari, Sandra S. Padula, A. Aleksandrov, G. Antchev, R. Hadjiiska, P. Iaydjiev, M. Misheva, M. Shopova, G. Sultanov, A. Dimitrov, L. Litov, B. Pavlov, P. Petkov, A. Petrov, E. Shumka, S. Keshri, S. Thakur, T. Cheng, T. Javaid, L. Yuan, Z. Hu, J. Liu, K. Yi, G. M. Chen, H. S. Chen, M. Chen, F. Iemmi, C. H. Jiang, A. Kapoor, H. Liao, Z.-A. Liu, R. Sharma, J. N. Song, J. Tao, C. Wang, J. Wang, Z. Wang, H. Zhang, A. Agapitos, Y. Ban, A. Levin, C. Li, Q. Li, Y. Mao, S. J. Qian, X. Sun, D. Wang, H. Yang, L. Zhang, C. Zhou, Z. You, N. Lu, G. Bauer, X. Gao, D. Leggat, H. Okawa, Z. Lin, C. Lu, M. Xiao, C. Avila, D. A. Barbosa Trujillo, A. Cabrera, C. Florez, J. Fraga, J. A. Reyes Vega, J. Mejia Guisao, F. Ramirez, M. Rodriguez, J. D. Ruiz Alvarez, D. Giljanovic, N. Godinovic, D. Lelas, A. Sculac, M. Kovac, T. Sculac, P. Bargassa, V. Brigljevic, B. K. Chitroda, D. Ferencek, S. Mishra, A. Starodumov, T. Susa, A. Attikis, K. Christoforou, S. Konstantinou, J. Mousa, C. Nicolaou, F. Ptochos, P. A. Razis, H. Rykaczewski, H. Saka, A. Stepennov, M. Finger, M. Finger, A. Kveton, E. Ayala, E. Carrera Jarrin, S. Elgammal, A. Ellithi Kamel, M. A. Mahmoud, Y. Mohammed, K. Ehataht, M. Kadastik, T. Lange, S. Nandan, C. Nielsen, J. Pata, M. Raidal, L. Tani, C. Veelken, H. Kirschenmann, K. Osterberg, M. Voutilainen, S. Bharthuar, E. Brücken, F. Garcia, K. T. S. Kallonen, R. Kinnunen, T. Lampén, K. Lassila-Perini, S. Lehti, T. Lindén, L. Martikainen, M. Myllymäki, M. M. Rantanen, H. Siikonen, E. Tuominen, J. Tuominiemi, P. Luukka, H. Petrow, M. Besancon, F. Couderc, M. Dejardin, D. Denegri, J. L. Faure, F. Ferri, S. Ganjour, P. Gras, G. Hamel de Monchenault, V. Lohezic, J. Malcles, J. Rander, A. Rosowsky, M. Ö. Sahin, A. Savoy-Navarro, P. Simkina, M. Titov, M. Tornago, C. Baldenegro Barrera, F. Beaudette, A. Buchot Perraguin, P. Busson, A. Cappati, C. Charlot, M. Chiusi, F. Damas, O. Davignon, A. De Wit, B. A. Fontana Santos Alves, S. Ghosh, A. Gilbert, R. Granier de Cassagnac, A. Hakimi, B. Harikrishnan, L. Kalipoliti, G. Liu, J. Motta, M. Nguyen, C. Ochando, L. Portales, R. Salerno, J. B. Sauvan, Y. Sirois, A. Tarabini, E. Vernazza, A. Zabi, A. Zghiche, J.-L. Agram, J. Andrea, D. Apparu, D. Bloch, J.-M. Brom, E. C. Chabert, C. Collard, S. Falke, U. Goerlach, C. Grimault, R. Haeberle, A.-C. Le Bihan, M. Meena, G. Saha, M. A. Sessini, P. Van Hove, S. Beauceron, B. Blancon, G. Boudoul, N. Chanon, J. Choi, D. Contardo, P. Depasse, C. Dozen, H. El Mamouni, J. Fay, S. Gascon, M. Gouzevitch, C. Greenberg, G. Grenier, B. Ille, I. B. Laktineh, M. Lethuillier, L. Mirabito, S. Perries, A. Purohit, M. Vander Donckt, P. Verdier, J. Xiao, G. Adamov, I. Lomidze, Z. Tsamalaidze, V. Botta, L. Feld, K. Klein, M. Lipinski, D. Meuser, A. Pauls, N. Röwert, M. Teroerde, S. Diekmann, A. Dodonova, N. Eich, D. Eliseev, F. Engelke, J. Erdmann, M. Erdmann, P. Fackeldey, B. Fischer, T. Hebbeker, K. Hoepfner, F. Ivone, A. Jung, M. Y. Lee, L. Mastrolorenzo, F. Mausolf, M. Merschmeyer, A. Meyer, S. Mukherjee, D. Noll, F. Nowotny, A. Pozdnyakov, Y. Rath, W. Redjeb, F. Rehm, H. Reithler, U. Sarkar, V. Sarkisovi, A. Schmidt, A. Sharma, J. L. Spah, A. Stein, F. Torres Da Silva De Araujo, L. Vigilante, S. Wiedenbeck, S. Zaleski, C. Dziwok, G. Flügge, W. Haj Ahmad, T. Kress, A. Nowack, O. Pooth, A. Stahl, T. Ziemons, A. Zotz, H. Aarup Petersen, M. Aldaya Martin, J. Alimena, S. Amoroso, Y. An, S. Baxter, M. Bayatmakou, H. Becerril Gonzalez, O. Behnke, A. Belvedere, S. Bhattacharya, F. Blekman, K. Borras, A. Campbell, A. Cardini, C. Cheng, F. Colombina, S. Consuegra Rodríguez, G. Correia Silva, M. De Silva, G. Eckerlin, D. Eckstein, L. I. Estevez Banos, O. Filatov, E. Gallo, A. Geiser, A. Giraldi, G. Greau, V. Guglielmi, M. Guthoff, A. Hinzmann, A. Jafari, L. Jeppe, N. Z. Jomhari, B. Kaech, M. Kasemann, C. Kleinwort, R. Kogler, M. Komm, D. Krücker, W. Lange, D. Leyva Pernia, K. Lipka, W. Lohmann, R. Mankel, I.-A. Melzer-Pellmann, M. Mendizabal Morentin, A. B. Meyer, G. Milella, A. Mussgiller, L. P. Nair, A. Nürnberg, Y. Otarid, J. Park, D. Pérez Adán, E. Ranken, A. Raspereza, B. Ribeiro Lopes, J. Rübenach, A. Saggio, M. Scham, S. Schnake, P. Schütze, C. Schwanenberger, D. Selivanova, K. Sharko, M. Shchedrolosiev, R. E. Sosa Ricardo, D. Stafford, F. Vazzoler, A. Ventura Barroso, R. Walsh, Q. Wang, Y. Wen, K. Wichmann, L. Wiens, C. Wissing, Y. Yang, A. Zimermmane Castro Santos, A. Albrecht, S. Albrecht, M. Antonello, S. Bein, L. Benato, S. Bollweg, M. Bonanomi, P. Connor, M. Eich, K. El Morabit, Y. Fischer, A. Fröhlich, C. Garbers, E. Garutti, A. Grohsjean, M. Hajheidari, J. Haller, H. R. Jabusch, G. Kasieczka, P. Keicher, R. Klanner, W. Korcari, T. Kramer, V. Kutzner, F. Labe, J. Lange, A. Lobanov, C. Matthies, A. Mehta, L. Moureaux, M. Mrowietz, A. Nigamova, Y. Nissan, A. Paasch, K. J. Pena Rodriguez, T. Quadfasel, B. Raciti, M. Rieger, D. Savoiu, J. Schindler, P. Schleper, M. Schröder, J. Schwandt, M. Sommerhalder, H. Stadie, G. Steinbrück, A. Tews, M. Wolf, S. Brommer, M. Burkart, E. Butz, T. Chwalek, A. Dierlamm, A. Droll, N. Faltermann, M. Giffels, A. Gottmann, F. Hartmann, R. Hofsaess, M. Horzela, U. Husemann, J. Kieseler, M. Klute, R. Koppenhöfer, J. M. Lawhorn, M. Link, A. Lintuluoto, S. Maier, S. Mitra, M. Mormile, Th. Müller, M. Neukum, M. Oh, M. Presilla, G. Quast, K. Rabbertz, B. Regnery, N. Shadskiy, I. Shvetsov, H. J. Simonis, M. Toms, N. Trevisani, R. Ulrich, R. F. Von Cube, M. Wassmer, S. Wieland, F. Wittig, R. Wolf, X. Zuo, G. Anagnostou, G. Daskalakis, A. Kyriakis, A. Papadopoulos, A. Stakia, P. Kontaxakis, G. Melachroinos, A. Panagiotou, I. Papavergou, I. Paraskevas, N. Saoulidou, K. Theofilatos, E. Tziaferi, K. Vellidis, I. Zisopoulos, G. Bakas, T. Chatzistavrou, G. Karapostoli, K. Kousouris, I. Papakrivopoulos, E. Siamarkou, G. Tsipolitis, A. Zacharopoulou, K. Adamidis, I. Bestintzanos, I. Evangelou, C. Foudas, C. Kamtsikis, P. Katsoulis, P. Kokkas, P. G. Kosmoglou Kioseoglou, N. Manthos, I. Papadopoulos, J. Strologas, M. Bartók, C. Hajdu, D. Horvath, K. Márton, F. Sikler, V. Veszpremi, M. Csanád, K. Farkas, M. M. A. Gadallah, Á. Kadlecsik, P. Major, K. Mandal, G. Pásztor, A. J. Rádl, G. I. Veres, P. Raics, B. Ujvari, G. Zilizi, G. Bencze, S. Czellar, J. Molnar, Z. Szillasi, T. Csorgo, F. Nemes, T. Novak, J. Babbar, S. Bansal, S. B. Beri, V. Bhatnagar, G. Chaudhary, S. Chauhan, N. Dhingra, A. Kaur, A. Kaur, H. Kaur, M. Kaur, S. Kumar, K. Sandeep, T. Sheokand, J. B. Singh, A. Singla, A. Ahmed, A. Bhardwaj, A. Chhetri, B. C. Choudhary, A. Kumar, A. Kumar, M. Naimuddin, K. Ranjan, S. Saumya, S. Baradia, S. Barman, S. Bhattacharya, S. Dutta, S. Dutta, S. Sarkar, M. M. Ameen, P. K. Behera, S. C. Behera, S. Chatterjee, P. Jana, P. Kalbhor, J. R. Komaragiri, D. Kumar, L. Panwar, P. R. Pujahari, N. R. Saha, A. Sharma, A. K. Sikdar, S. Verma, S. Dugad, M. Kumar, G. B. Mohanty, P. Suryadevara, A. Bala, S. Banerjee, R. M. Chatterjee, R. K. Dewanjee, M. Guchait, Sh. Jain, A. Jaiswal, S. Karmakar, S. Kumar, G. Majumder, K. Mazumdar, S. Parolia, A. Thachayath, S. Bahinipati, C. Kar, D. Maity, P. Mal, T. Mishra, V. K. Muraleedharan Nair Bindhu, K. Naskar, A. Nayak, P. Sadangi, P. Saha, S. K. Swain, S. Varghese, D. Vats, S. Acharya, A. Alpana, S. Dube, B. Gomber, B. Kansal, A. Laha, B. Sahu, S. Sharma, K. Y. Vaish, H. Bakhshiansohi, E. Khazaie, M. Zeinali, S. Chenarani, S. M. Etesami, M. Khakzad, M. Mohammadi Najafabadi, M. Grunewald, M. Abbrescia, R. Aly, A. Colaleo, D. Creanza, B. D’Anzi, N. De Filippis, M. De Palma, A. Di Florio, W. Elmetenawee, L. Fiore, G. Iaselli, M. Louka, G. Maggi, M. Maggi, I. Margjeka, V. Mastrapasqua, S. My, S. Nuzzo, A. Pellecchia, A. Pompili, G. Pugliese, R. Radogna, G. Ramirez-Sanchez, D. Ramos, A. Ranieri, L. Silvestris, F. M. Simone, Ü. Sözbilir, A. Stamerra, R. Venditti, P. Verwilligen, A. Zaza, G. Abbiendi, C. Battilana, D. Bonacorsi, L. Borgonovi, R. Campanini, P. Capiluppi, A. Castro, F. R. Cavallo, M. Cuffiani, G. M. Dallavalle, T. Diotalevi, F. Fabbri, A. Fanfani, D. Fasanella, P. Giacomelli, L. Giommi, C. Grandi, L. Guiducci, S. Lo Meo, L. Lunerti, G. Masetti, F. L. Navarria, A. Perrotta, F. Primavera, A. M. Rossi, T. Rovelli, G. P. Siroli, S. Costa, A. Di Mattia, R. Potenza, A. Tricomi, C. Tuve, P. Assiouras, G. Barbagli, G. Bardelli, B. Camaiani, A. Cassese, R. Ceccarelli, V. Ciulli, C. Civinini, R. D’Alessandro, E. Focardi, T. Kello, G. Latino, P. Lenzi, M. Lizzo, M. Meschini, S. Paoletti, A. Papanastassiou, G. Sguazzoni, L. Viliani, L. Benussi, S. Bianco, S. Meola, D. Piccolo, P. Chatagnon, F. Ferro, E. Robutti, S. Tosi, A. Benaglia, G. Boldrini, F. Brivio, F. Cetorelli, F. De Guio, M. E. Dinardo, P. Dini, S. Gennai, R. Gerosa, A. Ghezzi, P. Govoni, L. Guzzi, M. T. Lucchini, M. Malberti, S. Malvezzi, A. Massironi, D. Menasce, L. Moroni, M. Paganoni, D. Pedrini, B. S. Pinolini, S. Ragazzi, T. Tabarelli de Fatis, D. Zuolo, S. Buontempo, A. Cagnotta, F. Carnevali, N. Cavallo, F. Fabozzi, A. O. M. Iorio, L. Lista, P. Paolucci, B. Rossi, C. Sciacca, R. Ardino, P. Azzi, N. Bacchetta, D. Bisello, P. Bortignon, A. Bragagnolo, R. Carlin, P. Checchia, T. Dorigo, F. Gasparini, U. Gasparini, F. Gonella, E. Lusiani, M. Margoni, F. Marini, M. Migliorini, J. Pazzini, P. Ronchese, R. Rossin, F. Simonetto, G. Strong, M. Tosi, A. Triossi, S. Ventura, H. Yarar, M. Zanetti, P. Zotto, A. Zucchetta, G. Zumerle, S. Abu Zeid, C. Aimè, A. Braghieri, S. Calzaferri, D. Fiorina, P. Montagna, V. Re, C. Riccardi, P. Salvini, I. Vai, P. Vitulo, S. Ajmal, G. M. Bilei, D. Ciangottini, L. Fanò, M. Magherini, G. Mantovani, V. Mariani, M. Menichelli, F. Moscatelli, A. Rossi, A. Santocchia, D. Spiga, T. Tedeschi, P. Asenov, P. Azzurri, G. Bagliesi, R. Bhattacharya, L. Bianchini, T. Boccali, E. Bossini, D. Bruschini, R. Castaldi, M. A. Ciocci, M. Cipriani, V. D’Amante, R. Dell’Orso, S. Donato, A. Giassi, F. Ligabue, D. Matos Figueiredo, A. Messineo, M. Musich, F. Palla, A. Rizzi, G. Rolandi, S. Roy Chowdhury, T. Sarkar, A. Scribano, P. Spagnolo, R. Tenchini, G. Tonelli, N. Turini, A. Venturi, P. G. Verdini, P. Barria, M. Campana, F. Cavallari, L. Cunqueiro Mendez, D. Del Re, E. Di Marco, M. Diemoz, F. Errico, E. Longo, P. Meridiani, J. Mijuskovic, G. Organtini, F. Pandolfi, R. Paramatti, C. Quaranta, S. Rahatlou, C. Rovelli, F. Santanastasio, L. Soffi, N. Amapane, R. Arcidiacono, S. Argiro, M. Arneodo, N. Bartosik, R. Bellan, A. Bellora, C. Biino, C. Borca, N. Cartiglia, M. Costa, R. Covarelli, N. Demaria, L. Finco, M. Grippo, B. Kiani, F. Legger, F. Luongo, C. Mariotti, L. Markovic, S. Maselli, A. Mecca, E. Migliore, M. Monteno, R. Mulargia, M. M. Obertino, G. Ortona, L. Pacher, N. Pastrone, M. Pelliccioni, M. Ruspa, F. Siviero, V. Sola, A. Solano, A. Staiano, C. Tarricone, D. Trocino, G. Umoret, E. Vlasov, S. Belforte, V. Candelise, M. Casarsa, F. Cossutti, K. De Leo, G. Della Ricca, S. Dogra, J. Hong, C. Huh, B. Kim, D. H. Kim, J. Kim, H. Lee, S. W. Lee, C. S. Moon, Y. D. Oh, M. S. Ryu, S. Sekmen, Y. C. Yang, M. S. Kim, G. Bak, P. Gwak, H. Kim, D. H. Moon, E. Asilar, D. Kim, T. J. Kim, J. A. Merlin, S. Choi, S. Han, B. Hong, K. Lee, K. S. Lee, S. Lee, J. Park, S. K. Park, J. Yoo, J. Goh, S. Yang, H. S. Kim, Y. Kim, S. Lee, J. Almond, J. H. Bhyun, J. Choi, W. Jun, J. Kim, S. Ko, H. Kwon, H. Lee, J. Lee, J. Lee, B. H. Oh, S. B. Oh, H. Seo, U. K. Yang, I. Yoon, W. Jang, D. Y. Kang, Y. Kang, S. Kim, B. Ko, J. S. H. Lee, Y. Lee, I. C. Park, Y. Roh, I. J. Watson, S. Ha, H. D. Yoo, M. Choi, M. R. Kim, H. Lee, Y. Lee, I. Yu, T. Beyrouthy, Y. Maghrbi, K. Dreimanis, A. Gaile, G. Pikurs, A. Potrebko, M. Seidel, V. Veckalns, N. R. Strautnieks, M. Ambrozas, A. Juodagalvis, A. Rinkevicius, G. Tamulaitis, N. Bin Norjoharuddeen, I. Yusuff, Z. Zolkapli, J. F. Benitez, A. Castaneda Hernandez, H. A. Encinas Acosta, L. G. Gallegos Maríñez, M. León Coello, J. A. Murillo Quijada, A. Sehrawat, L. Valencia Palomo, G. Ayala, H. Castilla-Valdez, H. Crotte Ledesma, E. De La Cruz-Burelo, I. Heredia-De La Cruz, R. Lopez-Fernandez, C. A. Mondragon Herrera, A. Sánchez Hernández, C. Oropeza Barrera, M. Ramírez García, I. Bautista, I. Pedraza, H. A. Salazar Ibarguen, C. Uribe Estrada, I. Bubanja, N. Raicevic, P. H. Butler, A. Ahmad, M. I. Asghar, A. Awais, M. I. M. Awan, H. R. Hoorani, W. A. Khan, V. Avati, L. Grzanka, M. Malawski, H. Bialkowska, M. Bluj, B. Boimska, M. Górski, M. Kazana, M. Szleper, P. Zalewski, K. Bunkowski, K. Doroba, A. Kalinowski, M. Konecki, J. Krolikowski, A. Muhammad, K. Pozniak, W. Zabolotny, M. Araujo, D. Bastos, C. Beirão Da Cruz E Silva, A. Boletti, M. Bozzo, T. Camporesi, G. Da Molin, P. Faccioli, M. Gallinaro, J. Hollar, N. Leonardo, T. Niknejad, A. Petrilli, M. Pisano, J. Seixas, J. Varela, J. W. Wulff, P. Adzic, P. Milenovic, M. Dordevic, J. Milosevic, V. Rekovic, M. Aguilar-Benitez, J. Alcaraz Maestre, Cristina F. Bedoya, M. Cepeda, M. Cerrada, N. Colino, B. De La Cruz, A. Delgado Peris, A. Escalante Del Valle, D. Fernández Del Val, J. P. Fernández Ramos, J. Flix, M. C. Fouz, O. Gonzalez Lopez, S. Goy Lopez, J. M. Hernandez, M. I. Josa, D. Moran, C. M. Morcillo Perez, Á. Navarro Tobar, C. Perez Dengra, A. Pérez-Calero Yzquierdo, J. Puerta Pelayo, I. Redondo, D. D. Redondo Ferrero, L. Romero, S. Sánchez Navas, L. Urda Gómez, J. Vazquez Escobar, C. Willmott, J. F. de Trocóniz, B. Alvarez Gonzalez, J. Cuevas, J. Fernandez Menendez, S. Folgueras, I. Gonzalez Caballero, J. R. González Fernández, E. Palencia Cortezon, C. Ramón Álvarez, V. Rodríguez Bouza, A. Soto Rodríguez, A. Trapote, C. Vico Villalba, P. Vischia, S. Bhowmik, S. Blanco Fernández, J. A. Brochero Cifuentes, I. J. Cabrillo, A. Calderon, J. Duarte Campderros, M. Fernandez, G. Gomez, C. Lasaosa García, C. Martinez Rivero, P. Martinez Ruiz del Arbol, F. Matorras, P. Matorras Cuevas, E. Navarrete Ramos, J. Piedra Gomez, L. Scodellaro, I. Vila, J. M. Vizan Garcia, M. K. Jayananda, B. Kailasapathy, D. U. J. Sonnadara, D. D. C. Wickramarathna, W. G. D. Dharmaratna, K. Liyanage, N. Perera, N. Wickramage, D. Abbaneo, C. Amendola, E. Auffray, G. Auzinger, J. Baechler, D. Barney, A. Bermúdez Martínez, M. Bianco, B. Bilin, A. A. Bin Anuar, A. Bocci, C. Botta, E. Brondolin, C. Caillol, G. Cerminara, N. Chernyavskaya, D. d’Enterria, A. Dabrowski, A. David, A. De Roeck, M. M. Defranchis, M. Deile, M. Dobson, L. Forthomme, G. Franzoni, W. Funk, S. Giani, D. Gigi, K. Gill, F. Glege, L. Gouskos, M. Haranko, J. Hegeman, B. Huber, V. Innocente, T. James, P. Janot, S. Laurila, P. Lecoq, E. Leutgeb, C. Lourenço, B. Maier, L. Malgeri, M. Mannelli, A. C. Marini, M. Matthewman, F. Meijers, S. Mersi, E. Meschi, V. Milosevic, F. Monti, F. Moortgat, M. Mulders, I. Neutelings, S. Orfanelli, F. Pantaleo, G. Petrucciani, A. Pfeiffer, M. Pierini, D. Piparo, H. Qu, D. Rabady, G. Reales Gutiérrez, M. Rovere, H. Sakulin, S. Scarfi, C. Schwick, M. Selvaggi, A. Sharma, K. Shchelina, P. Silva, P. Sphicas, A. G. Stahl Leiton, A. Steen, S. Summers, D. Treille, P. Tropea, A. Tsirou, D. Walter, J. Wanczyk, J. Wang, S. Wuchterl, P. Zehetner, P. Zejdl, W. D. Zeuner, T. Bevilacqua, L. Caminada, A. Ebrahimi, W. Erdmann, R. Horisberger, Q. Ingram, H. C. Kaestli, D. Kotlinski, C. Lange, M. Missiroli, L. Noehte, T. Rohe, T. K. Aarrestad, K. Androsov, M. Backhaus, A. Calandri, C. Cazzaniga, K. Datta, A. De Cosa, G. Dissertori, M. Dittmar, M. Donegà, F. Eble, M. Galli, K. Gedia, F. Glessgen, C. Grab, D. Hits, W. Lustermann, A.-M. Lyon, R. A. Manzoni, M. Marchegiani, L. Marchese, C. Martin Perez, A. Mascellani, F. Nessi-Tedaldi, F. Pauss, V. Perovic, S. Pigazzini, C. Reissel, T. Reitenspiess, B. Ristic, F. Riti, D. Ruini, R. Seidita, J. Steggemann, D. Valsecchi, R. Wallny, C. Amsler, P. Bärtschi, D. Brzhechko, M. F. Canelli, K. Cormier, J. K. Heikkilä, M. Huwiler, W. Jin, A. Jofrehei, B. Kilminster, S. Leontsinis, S. P. Liechti, A. Macchiolo, P. Meiring, U. Molinatti, A. Reimers, P. Robmann, S. Sanchez Cruz, M. Senger, Y. Takahashi, R. Tramontano, C. Adloff, D. Bhowmik, C. M. Kuo, W. Lin, P. K. Rout, P. C. Tiwari, S. S. Yu, L. Ceard, Y. Chao, K. F. Chen, P. S. Chen, Z. G. Chen, A. De Iorio, W.-S. Hou, T. H. Hsu, Y. W. Kao, R. Khurana, G. Kole, Y. Y. Li, R.-S. Lu, E. Paganis, X. F. Su, J. Thomas-Wilsker, L. S. Tsai, H. Y. Wu, E. Yazgan, C. Asawatangtrakuldee, N. Srimanobhas, V. Wachirapusitanand, D. Agyel, F. Boran, Z. S. Demiroglu, F. Dolek, I. Dumanoglu, E. Eskut, Y. Guler, E. Gurpinar Guler, C. Isik, O. Kara, A. Kayis Topaksu, U. Kiminsu, G. Onengut, K. Ozdemir, A. Polatoz, B. Tali, U. G. Tok, S. Turkcapar, E. Uslan, I. S. Zorbakir, M. Yalvac, B. Akgun, I. O. Atakisi, E. Gülmez, M. Kaya, O. Kaya, S. Tekten, A. Cakir, K. Cankocak, Y. Komurcu, S. Sen, O. Aydilek, S. Cerci, V. Epshteyn, B. Hacisahinoglu, I. Hos, B. Kaynak, S. Ozkorucuklu, O. Potok, H. Sert, C. Simsek, C. Zorbilmez, B. Isildak, D. Sunar Cerci, A. Boyaryntsev, B. Grynyov, L. Levchuk, D. Anthony, J. J. Brooke, A. Bundock, F. Bury, E. Clement, D. Cussans, H. Flacher, M. Glowacki, J. Goldstein, H. F. Heath, L. Kreczko, S. Paramesvaran, S. Seif El Nasr-Storey, V. J. Smith, N. Stylianou, K. Walkingshaw Pass, R. White, A. H. Ball, K. W. Bell, A. Belyaev, C. Brew, R. M. Brown, D. J. A. Cockerill, C. Cooke, K. V. Ellis, K. Harder, S. Harper, M.-L. Holmberg, J. Linacre, K. Manolopoulos, D. M. Newbold, E. Olaiya, D. Petyt, T. Reis, G. Salvi, T. Schuh, C. H. Shepherd-Themistocleous, I. R. Tomalin, T. Williams, R. Bainbridge, P. Bloch, C. E. Brown, O. Buchmuller, V. Cacchio, C. A. Carrillo Montoya, G. S. Chahal, D. Colling, J. S. Dancu, I. Das, P. Dauncey, G. Davies, J. Davies, M. Della Negra, S. Fayer, G. Fedi, G. Hall, M. H. Hassanshahi, A. Howard, G. Iles, M. Knight, J. Langford, J. León Holgado, L. Lyons, A.-M. Magnan, S. Malik, M. Mieskolainen, J. Nash, M. Pesaresi, B. C. Radburn-Smith, A. Richards, A. Rose, K. Savva, C. Seez, R. Shukla, A. Tapper, K. Uchida, G. P. Uttley, L. H. Vage, T. Virdee, M. Vojinovic, N. Wardle, D. Winterbottom, K. Coldham, J. E. Cole, A. Khan, P. Kyberd, I. D. Reid, S. Abdullin, A. Brinkerhoff, B. Caraway, J. Dittmann, K. Hatakeyama, J. Hiltbrand, B. McMaster, M. Saunders, S. Sawant, C. Sutantawibul, J. Wilson, R. Bartek, A. Dominguez, C. Huerta Escamilla, A. E. Simsek, R. Uniyal, A. M. Vargas Hernandez, B. Bam, R. Chudasama, S. I. Cooper, S. V. Gleyzer, C. U. Perez, P. Rumerio, E. Usai, R. Yi, A. Akpinar, D. Arcaro, C. Cosby, Z. Demiragli, C. Erice, C. Fangmeier, C. Fernandez Madrazo, E. Fontanesi, D. Gastler, F. Golf, S. Jeon, I. Reed, J. Rohlf, K. Salyer, D. Sperka, D. Spitzbart, I. Suarez, A. Tsatsos, S. Yuan, A. G. Zecchinelli, G. Benelli, X. Coubez, D. Cutts, M. Hadley, U. Heintz, J. M. Hogan, T. Kwon, G. Landsberg, K. T. Lau, D. Li, J. Luo, S. Mondal, M. Narain, N. Pervan, S. Sagir, F. Simpson, M. Stamenkovic, W. Y. Wong, X. Yan, W. Zhang, S. Abbott, J. Bonilla, C. Brainerd, R. Breedon, M. Calderon De La Barca Sanchez, M. Chertok, M. Citron, J. Conway, P. T. Cox, R. Erbacher, F. Jensen, O. Kukral, G. Mocellin, M. Mulhearn, D. Pellett, W. Wei, Y. Yao, F. Zhang, M. Bachtis, R. Cousins, A. Datta, G. Flores Avila, J. Hauser, M. Ignatenko, M. A. Iqbal, T. Lam, E. Manca, A. Nunez Del Prado, D. Saltzberg, V. Valuev, R. Clare, J. W. Gary, M. Gordon, G. Hanson, W. Si, S. Wimpenny, J. G. Branson, S. Cittolin, S. Cooperstein, D. Diaz, J. Duarte, L. Giannini, J. Guiang, R. Kansal, V. Krutelyov, R. Lee, J. Letts, M. Masciovecchio, F. Mokhtar, S. Mukherjee, M. Pieri, M. Quinnan, B. V. Sathia Narayanan, V. Sharma, M. Tadel, E. Vourliotis, F. Würthwein, Y. Xiang, A. Yagil, A. Barzdukas, L. Brennan, C. Campagnari, A. Dorsett, J. Incandela, J. Kim, A. J. Li, P. Masterson, H. Mei, J. Richman, U. Sarica, R. Schmitz, F. Setti, J. Sheplock, D. Stuart, T.Á. Vámi, S. Wang, A. Bornheim, O. Cerri, A. Latorre, J. Mao, H. B. Newman, M. Spiropulu, J. R. Vlimant, C. Wang, S. Xie, R. Y. Zhu, J. Alison, S. An, M. B. Andrews, P. Bryant, M. Cremonesi, V. Dutta, T. Ferguson, A. Harilal, C. Liu, T. Mudholkar, S. Murthy, P. Palit, M. Paulini, A. Roberts, A. Sanchez, W. Terrill, J. P. Cumalat, W. T. Ford, A. Hart, A. Hassani, G. Karathanasis, E. MacDonald, N. Manganelli, A. Perloff, C. Savard, N. Schonbeck, K. Stenson, K. A. Ulmer, S. R. Wagner, N. Zipper, J. Alexander, S. Bright-Thonney, X. Chen, D. J. Cranshaw, J. Fan, X. Fan, D. Gadkari, S. Hogan, P. Kotamnives, J. Monroy, M. Oshiro, J. R. Patterson, J. Reichert, M. Reid, A. Ryd, J. Thom, P. Wittich, R. Zou, M. Albrow, M. Alyari, O. Amram, G. Apollinari, A. Apresyan, L. A. T. Bauerdick, D. Berry, J. Berryhill, P. C. Bhat, K. Burkett, J. N. Butler, A. Canepa, G. B. Cerati, H. W. K. Cheung, F. Chlebana, G. Cummings, J. Dickinson, I. Dutta, V. D. Elvira, Y. Feng, J. Freeman, A. Gandrakota, Z. Gecse, L. Gray, D. Green, A. Grummer, S. Grünendahl, D. Guerrero, O. Gutsche, R. M. Harris, R. Heller, T. C. Herwig, J. Hirschauer, L. Horyn, B. Jayatilaka, S. Jindariani, M. Johnson, U. Joshi, T. Klijnsma, B. Klima, K. H. M. Kwok, S. Lammel, D. Lincoln, R. Lipton, T. Liu, C. Madrid, K. Maeshima, C. Mantilla, D. Mason, P. McBride, P. Merkel, S. Mrenna, S. Nahn, J. Ngadiuba, D. Noonan, V. Papadimitriou, N. Pastika, K. Pedro, C. Pena, F. Ravera, A. Reinsvold Hall, L. Ristori, E. Sexton-Kennedy, N. Smith, A. Soha, L. Spiegel, S. Stoynev, J. Strait, L. Taylor, S. Tkaczyk, N. V. Tran, L. Uplegger, E. W. Vaandering, I. Zoi, C. Aruta, P. Avery, D. Bourilkov, L. Cadamuro, P. Chang, V. Cherepanov, R. D. Field, E. Koenig, M. Kolosova, J. Konigsberg, A. Korytov, K. H. Lo, K. Matchev, N. Menendez, G. Mitselmakher, K. Mohrman, A. Muthirakalayil Madhu, N. Rawal, D. Rosenzweig, S. Rosenzweig, K. Shi, J. Wang, T. Adams, A. Al Kadhim, A. Askew, S. Bower, R. Habibullah, V. Hagopian, R. Hashmi, R. S. Kim, S. Kim, T. Kolberg, G. Martinez, H. Prosper, P. R. Prova, M. Wulansatiti, R. Yohay, J. Zhang, B. Alsufyani, M. M. Baarmand, S. Butalla, T. Elkafrawy, M. Hohlmann, R. Kumar Verma, M. Rahmani, E. Yanes, M. R. Adams, A. Baty, C. Bennett, R. Cavanaugh, R. Escobar Franco, O. Evdokimov, C. E. Gerber, D. J. Hofman, J. H. Lee, D. S. Lemos, A. H. Merrit, C. Mills, S. Nanda, G. Oh, B. Ozek, D. Pilipovic, R. Pradhan, T. Roy, S. Rudrabhatla, M. B. Tonjes, N. Varelas, Z. Ye, J. Yoo, M. Alhusseini, D. Blend, K. Dilsiz, L. Emediato, G. Karaman, O. K. Köseyan, J.-P. Merlo, A. Mestvirishvili, J. Nachtman, O. Neogi, H. Ogul, Y. Onel, A. Penzo, C. Snyder, E. Tiras, B. Blumenfeld, L. Corcodilos, J. Davis, A. V. Gritsan, L. Kang, S. Kyriacou, P. Maksimovic, M. Roguljic, J. Roskes, S. Sekhar, M. Swartz, A. Abreu, L. F. Alcerro Alcerro, J. Anguiano, P. Baringer, A. Bean, Z. Flowers, D. Grove, J. King, G. Krintiras, M. Lazarovits, C. Le Mahieu, C. Lindsey, J. Marquez, N. Minafra, M. Murray, M. Nickel, M. Pitt, S. Popescu, C. Rogan, C. Royon, R. Salvatico, S. Sanders, C. Smith, Q. Wang, G. Wilson, B. Allmond, A. Ivanov, K. Kaadze, A. Kalogeropoulos, D. Kim, Y. Maravin, K. Nam, J. Natoli, D. Roy, G. Sorrentino, F. Rebassoo, D. Wright, A. Baden, A. Belloni, Y. M. Chen, S. C. Eno, N. J. Hadley, S. Jabeen, R. G. Kellogg, T. Koeth, Y. Lai, S. Lascio, A. C. Mignerey, S. Nabili, C. Palmer, C. Papageorgakis, M. M. Paranjpe, L. Wang, J. Bendavid, I. A. Cali, M. D’Alfonso, J. Eysermans, C. Freer, G. Gomez-Ceballos, M. Goncharov, G. Grosso, P. Harris, D. Hoang, D. Kovalskyi, J. Krupa, L. Lavezzo, Y.-J. Lee, K. Long, C. Mironov, A. Novak, C. Paus, D. Rankin, C. Roland, G. Roland, S. Rothman, G. S. F. Stephans, Z. Wang, B. Wyslouch, T. J. Yang, B. Crossman, B. M. Joshi, C. Kapsiak, M. Krohn, D. Mahon, J. Mans, B. Marzocchi, S. Pandey, M. Revering, R. Rusack, R. Saradhy, N. Schroeder, N. Strobbe, M. A. Wadud, L. M. Cremaldi, K. Bloom, D. R. Claes, G. Haza, J. Hossain, C. Joo, I. Kravchenko, J. E. Siado, W. Tabb, A. Vagnerini, A. Wightman, F. Yan, D. Yu, H. Bandyopadhyay, L. Hay, I. Iashvili, A. Kharchilava, M. Morris, D. Nguyen, S. Rappoccio, H. Rejeb Sfar, A. Williams, G. Alverson, E. Barberis, J. Dervan, Y. Haddad, Y. Han, A. Krishna, J. Li, M. Lu, G. Madigan, R. Mccarthy, D. M. Morse, V. Nguyen, T. Orimoto, A. Parker, L. Skinnari, A. Tishelman-Charny, B. Wang, D. Wood, S. Bhattacharya, J. Bueghly, Z. Chen, S. Dittmer, K. A. Hahn, Y. Liu, Y. Miao, D. G. Monk, M. H. Schmitt, A. Taliercio, M. Velasco, G. Agarwal, R. Band, R. Bucci, S. Castells, A. Das, R. Goldouzian, M. Hildreth, K. W. Ho, K. Hurtado Anampa, T. Ivanov, C. Jessop, K. Lannon, J. Lawrence, N. Loukas, L. Lutton, J. Mariano, N. Marinelli, I. Mcalister, T. McCauley, C. Mcgrady, C. Moore, Y. Musienko, H. Nelson, M. Osherson, A. Piccinelli, R. Ruchti, A. Townsend, Y. Wan, M. Wayne, H. Yockey, M. Zarucki, L. Zygala, A. Basnet, B. Bylsma, M. Carrigan, L. S. Durkin, C. Hill, M. Joyce, M. Nunez Ornelas, K. Wei, B. L. Winer, B. R. Yates, F. M. Addesa, H. Bouchamaoui, P. Das, G. Dezoort, P. Elmer, A. Frankenthal, B. Greenberg, N. Haubrich, G. Kopp, S. Kwan, D. Lange, A. Loeliger, D. Marlow, I. Ojalvo, J. Olsen, A. Shevelev, D. Stickland, C. Tully, S. Malik, A. S. Bakshi, V. E. Barnes, S. Chandra, R. Chawla, S. Das, A. Gu, L. Gutay, M. Jones, A. W. Jung, D. Kondratyev, A. M. Koshy, M. Liu, G. Negro, N. Neumeister, G. Paspalaki, S. Piperov, V. Scheurer, J. F. Schulte, M. Stojanovic, J. Thieman, A. K. Virdi, F. Wang, W. Xie, J. Dolen, N. Parashar, A. Pathak, D. Acosta, T. Carnahan, K. M. Ecklund, P. J. Fernández Manteca, S. Freed, P. Gardner, F. J. M. Geurts, W. Li, O. Miguel Colin, B. P. Padley, R. Redjimi, J. Rotter, E. Yigitbasi, Y. Zhang, A. Bodek, P. de Barbaro, R. Demina, J. L. Dulemba, A. Garcia-Bellido, O. Hindrichs, A. Khukhunaishvili, N. Parmar, P. Parygin, E. Popova, R. Taus, K. Goulianos, B. Chiarito, J. P. Chou, Y. Gershtein, E. Halkiadakis, M. Heindl, D. Jaroslawski, O. Karacheban, I. Laflotte, A. Lath, R. Montalvo, K. Nash, H. Routray, S. Salur, S. Schnetzer, S. Somalwar, R. Stone, S. A. Thayil, S. Thomas, J. Vora, H. Wang, H. Acharya, D. Ally, A. G. Delannoy, S. Fiorendi, S. Higginbotham, T. Holmes, A. R. Kanuganti, N. Karunarathna, L. Lee, E. Nibigira, S. Spanier, D. Aebi, M. Ahmad, O. Bouhali, R. Eusebi, J. Gilmore, T. Huang, T. Kamon, H. Kim, S. Luo, R. Mueller, D. Overton, D. Rathjens, A. Safonov, N. Akchurin, J. Damgov, V. Hegde, A. Hussain, Y. Kazhykarim, K. Lamichhane, S. W. Lee, A. Mankel, T. Peltola, I. Volobouev, A. Whitbeck, E. Appelt, Y. Chen, S. Greene, A. Gurrola, W. Johns, R. Kunnawalkam Elayavalli, A. Melo, F. Romeo, P. Sheldon, S. Tuo, J. Velkovska, J. Viinikainen, B. Cardwell, B. Cox, J. Hakala, R. Hirosky, A. Ledovskoy, C. Neu, C. E. Perez Lara, P. E. Karchin, A. Aravind, S. Banerjee, K. Black, T. Bose, S. Dasu, I. De Bruyn, P. Everaerts, C. Galloni, H. He, M. Herndon, A. Herve, C. K. Koraka, A. Lanaro, R. Loveless, J. Madhusudanan Sreekala, A. Mallampalli, A. Mohammadi, S. Mondal, G. Parida, D. Pinna, A. Savin, V. Shang, V. Sharma, W. H. Smith, D. Teague, H. F. Tsoi, W. Vetens, A. Warden, S. Afanasiev, V. Andreev, Yu. Andreev, T. Aushev, M. Azarkin, A. Babaev, A. Belyaev, V. Blinov, E. Boos, V. Borshch, D. Budkouski, V. Chekhovsky, R. Chistov, M. Danilov, A. Dermenev, T. Dimova, D. Druzhkin, M. Dubinin, L. Dudko, A. Ershov, G. Gavrilov, V. Gavrilov, S. Gninenko, V. Golovtcov, N. Golubev, I. Golutvin, I. Gorbunov, A. Gribushin, Y. Ivanov, V. Kachanov, V. Karjavine, A. Karneyeu, V. Kim, M. Kirakosyan, D. Kirpichnikov, M. Kirsanov, V. Klyukhin, O. Kodolova, V. Korenkov, A. Kozyrev, N. Krasnikov, A. Lanev, P. Levchenko, N. Lychkovskaya, V. Makarenko, A. Malakhov, V. Matveev, V. Murzin, A. Nikitenko, S. Obraztsov, V. Oreshkin, V. Palichik, V. Perelygin, S. Petrushanko, S. Polikarpov, V. Popov, O. Radchenko, M. Savina, V. Savrin, V. Shalaev, S. Shmatov, S. Shulha, Y. Skovpen, S. Slabospitskii, V. Smirnov, A. Snigirev, D. Sosnov, V. Sulimov, E. Tcherniaev, A. Terkulov, O. Teryaev, I. Tlisova, A. Toropin, L. Uvarov, A. Uzunian, A. Vorobyev, N. Voytishin, B. S. Yuldashev, A. Zarubin, I. Zhizhin, A. Zhokin

**Affiliations:** 1 Yerevan Physics Institute, Yerevan, Armenia; 2 Institut für Hochenergiephysik, Vienna, Austria; 3 Universiteit Antwerpen, Antwerp, Belgium; 4 Vrije Universiteit Brussel, Brussels, Belgium; 5 Université Libre de Bruxelles, Brussels, Belgium; 6 Ghent University, Ghent, Belgium; 7 Université Catholique de Louvain, Louvain-la-Neuve, Belgium; 8 Centro Brasileiro de Pesquisas Fisicas, Rio de Janeiro, Brazil; 9 Universidade do Estado do Rio de Janeiro, Rio de Janeiro, Brazil; 10 Universidade Estadual Paulista, Universidade Federal do ABC, São Paulo, Brazil; 11 Institute for Nuclear Research and Nuclear Energy, Bulgarian Academy of Sciences, Sofia, Bulgaria; 12 University of Sofia, Sofia, Bulgaria; 13 Instituto De Alta Investigación, Universidad de Tarapacá, Casilla 7 D, Arica, Chile; 14 Beihang University, Beijing, China; 15 Department of Physics, Tsinghua University, Beijing, China; 16 Institute of High Energy Physics, Beijing, China; 17 State Key Laboratory of Nuclear Physics and Technology, Peking University, Beijing, China; 18 Sun Yat-Sen University, Guangzhou, China; 19 University of Science and Technology of China, Hefei, China; 20 Nanjing Normal University, Nanjing, China; 21 Institute of Modern Physics and Key Laboratory of Nuclear Physics and Ion-beam Application (MOE)-Fudan University, Shanghai, China; 22 Zhejiang University, Hangzhou, Zhejiang China; 23 Universidad de Los Andes, Bogotá, Colombia; 24 Universidad de Antioquia, Medellín, Colombia; 25 Faculty of Electrical Engineering, Mechanical Engineering and Naval Architecture, University of Split, Split, Croatia; 26 Faculty of Science, University of Split, Split, Croatia; 27 Institute Rudjer Boskovic, Zagreb, Croatia; 28 University of Cyprus, Nicosia, Cyprus; 29 Charles University, Prague, Czech Republic; 30 Escuela Politecnica Nacional, Quito, Ecuador; 31 Universidad San Francisco de Quito, Quito, Ecuador; 32 Academy of Scientific Research and Technology of the Arab Republic of Egypt, Egyptian Network of High Energy Physics, Cairo, Egypt; 33 Center for High Energy Physics (CHEP-FU), Fayoum University, El-Fayoum, Egypt; 34 National Institute of Chemical Physics and Biophysics, Tallinn, Estonia; 35 Department of Physics, University of Helsinki, Helsinki, Finland; 36 Helsinki Institute of Physics, Helsinki, Finland; 37 Lappeenranta-Lahti University of Technology, Lappeenranta, Finland; 38 IRFU, CEA, Université Paris-Saclay, Gif-sur-Yvette, France; 39 Laboratoire Leprince-Ringuet, CNRS/IN2P3, Ecole Polytechnique, Institut Polytechnique de Paris, Palaiseau, France; 40 Université de Strasbourg, CNRS, IPHC UMR 7178, Strasbourg, France; 41 Institut de Physique des 2 Infinis de Lyon (IP2I ), Villeurbanne, France; 42 Georgian Technical University, Tbilisi, Georgia; 43 I. Physikalisches Institut, RWTH Aachen University, Aachen, Germany; 44 RWTH Aachen University, III. Physikalisches Institut A, Aachen, Germany; 45 RWTH Aachen University, III. Physikalisches Institut B, Aachen, Germany; 46 Deutsches Elektronen-Synchrotron, Hamburg, Germany; 47 University of Hamburg, Hamburg, Germany; 48 Karlsruher Institut fuer Technologie, Karlsruhe, Germany; 49 Institute of Nuclear and Particle Physics (INPP), NCSR Demokritos, Agia Paraskevi, Greece; 50 National and Kapodistrian University of Athens, Athens, Greece; 51 National Technical University of Athens, Athens, Greece; 52 University of Ioánnina, Ioannina, Greece; 53 HUN-REN Wigner Research Centre for Physics, Budapest, Hungary; 54 MTA-ELTE Lendület CMS Particle and Nuclear Physics Group, Eötvös Loránd University, Budapest, Hungary; 55 Faculty of Informatics, University of Debrecen, Debrecen, Hungary; 56 Institute of Nuclear Research ATOMKI, Debrecen, Hungary; 57 MATE Institute of Technology, Karoly Robert Campus, Gyongyos, Hungary; 58 Panjab University, Chandigarh, India; 59 University of Delhi, Delhi, India; 60 Saha Institute of Nuclear Physics, HBNI, Kolkata, India; 61 Indian Institute of Technology Madras, Chennai, India; 62 Tata Institute of Fundamental Research-A, Mumbai, India; 63 Tata Institute of Fundamental Research-B, Mumbai, India; 64 National Institute of Science Education and Research, An OCC of Homi Bhabha National Institute, Bhubaneswar, Odisha India; 65 Indian Institute of Science Education and Research (IISER), Pune, India; 66 Isfahan University of Technology, Isfahan, Iran; 67 Institute for Research in Fundamental Sciences (IPM), Tehran, Iran; 68 University College Dublin, Dublin, Ireland; 69 INFN Sezione di Bari, Università di Bari, Politecnico di Bari, Bari, Italy; 70 INFN Sezione di Bologna, Università di Bologna, Bologna, Italy; 71 INFN Sezione di Catania, Università di Catania, Catania, Italy; 72 INFN Sezione di Firenze, Università di Firenze, Florence, Italy; 73 INFN Laboratori Nazionali di Frascati, Frascati, Italy; 74 INFN Sezione di Genova, Università di Genova, Genoa, Italy; 75 INFN Sezione di Milano-Bicocca, Università di Milano-Bicocca, Milan, Italy; 76 INFN Sezione di Napoli, Università di Napoli ‘Federico II’, Naples, Italy; Università della Basilicata, Potenza, Italy; Scuola Superiore Meridionale (SSM), Naples, Italy; 77 INFN Sezione di Padova, Università di Padova, Padova, Italy; Università di Trento, Trento, Italy; 78 INFN Sezione di Pavia, Università di Pavia, Pavia, Italy; 79 INFN Sezione di Perugia, Università di Perugia, Perugia, Italy; 80 INFN Sezione di Pisa, Università di Pisa, Scuola Normale Superiore di Pisa, Pisa, Italy; Università di Siena, Siena, Italy; 81 INFN Sezione di Roma, Sapienza Università di Roma, Rome, Italy; 82 INFN Sezione di Torino, Università di Torino, Turin, Italy; Università del Piemonte Orientale, Novara, Italy; 83 INFN Sezione di Trieste, Università di Trieste, Trieste, Italy; 84 Kyungpook National University, Daegu, Korea; 85 Department of Mathematics and Physics, GWNU, Gangneung, Korea; 86 Institute for Universe and Elementary Particles, Chonnam National University, Kwangju, Korea; 87 Hanyang University, Seoul, Korea; 88 Korea University, Seoul, Korea; 89 Department of Physics, Kyung Hee University, Seoul, Korea; 90 Sejong University, Seoul, Korea; 91 Seoul National University, Seoul, Korea; 92 University of Seoul, Seoul, Korea; 93 Department of Physics, Yonsei University, Seoul, Korea; 94 Sungkyunkwan University, Suwon, Korea; 95 College of Engineering and Technology, American University of the Middle East (AUM), Dasman, Kuwait; 96 Riga Technical University, Riga, Latvia; 97 University of Latvia (LU), Riga, Latvia; 98 Vilnius University, Vilnius, Lithuania; 99 National Centre for Particle Physics, Universiti Malaya, Kuala Lumpur, Malaysia; 100 Universidad de Sonora (UNISON), Hermosillo, Mexico; 101 Centro de Investigacion y de Estudios Avanzados del IPN, Mexico City, Mexico; 102 Universidad Iberoamericana, Mexico City, Mexico; 103 Benemerita Universidad Autonoma de Puebla, Puebla, Mexico; 104 University of Montenegro, Podgorica, Montenegro; 105 University of Canterbury, Christchurch, New Zealand; 106 National Centre for Physics, Quaid-I-Azam University, Islamabad, Pakistan; 107 Faculty of Computer Science, Electronics and Telecommunications, AGH University of Krakow, Kraków, Poland; 108 National Centre for Nuclear Research, Swierk, Poland; 109 Institute of Experimental Physics, Faculty of Physics, University of Warsaw, Warsaw, Poland; 110 Warsaw University of Technology, Warsaw, Poland; 111 Laboratório de Instrumentação e Física Experimental de Partículas, Lisbon, Portugal; 112 Faculty of Physics, University of Belgrade, Belgrade, Serbia; 113 VINCA Institute of Nuclear Sciences, University of Belgrade, Belgrade, Serbia; 114 Centro de Investigaciones Energéticas Medioambientales y Tecnológicas (CIEMAT), Madrid, Spain; 115 Universidad Autónoma de Madrid, Madrid, Spain; 116 Instituto Universitario de Ciencias y Tecnologías Espaciales de Asturias (ICTEA), Universidad de Oviedo, Oviedo, Spain; 117 Instituto de Física de Cantabria (IFCA), CSIC-Universidad de Cantabria, Santander, Spain; 118 University of Colombo, Colombo, Sri Lanka; 119 Department of Physics, University of Ruhuna, Matara, Sri Lanka; 120 CERN, European Organization for Nuclear Research, Geneva, Switzerland; 121 Paul Scherrer Institut, Villigen, Switzerland; 122 ETH Zurich-Institute for Particle Physics and Astrophysics (IPA), Zurich, Switzerland; 123 Universität Zürich, Zurich, Switzerland; 124 National Central University, Chung-Li, Taiwan; 125 National Taiwan University (NTU), Taipei, Taiwan; 126 High Energy Physics Research Unit, Department of Physics, Faculty of Science, Chulalongkorn University, Bangkok, Thailand; 127 Physics Department, Science and Art Faculty, Çukurova University, Adana, Turkey; 128 Physics Department, Middle East Technical University, Ankara, Turkey; 129 Bogazici University, Istanbul, Turkey; 130 Istanbul Technical University, Istanbul, Turkey; 131 Istanbul University, Istanbul, Turkey; 132 Yildiz Technical University, Istanbul, Turkey; 133 Institute for Scintillation Materials of National Academy of Science of Ukraine, Kharkiv, Ukraine; 134 National Science Centre, Kharkiv Institute of Physics and Technology, Kharkiv, Ukraine; 135 University of Bristol, Bristol, UK; 136 Rutherford Appleton Laboratory, Didcot, UK; 137 Imperial College, London, UK; 138 Brunel University, Uxbridge, UK; 139 Baylor University, Waco, TX USA; 140 Catholic University of America, Washington, DC USA; 141 The University of Alabama, Tuscaloosa, AL USA; 142 Boston University, Boston, MA USA; 143 Brown University, Providence, RI USA; 144 University of California, Davis, Davis, CA USA; 145 University of California, Los Angeles, CA USA; 146 University of California, Riverside, Riverside, CA USA; 147 University of California, San Diego, La Jolla, CA USA; 148 Department of Physics, University of California, Santa Barbara, Santa Barbara, CA USA; 149 California Institute of Technology, Pasadena, CA USA; 150 Carnegie Mellon University, Pittsburgh, PA USA; 151 University of Colorado Boulder, Boulder, CO USA; 152 Cornell University, Ithaca, NY USA; 153 Fermi National Accelerator Laboratory, Batavia, IL USA; 154 University of Florida, Gainesville, FL USA; 155 Florida State University, Tallahassee, FL USA; 156 Florida Institute of Technology, Melbourne, FL USA; 157 University of Illinois Chicago, Chicago, USA; 158 The University of Iowa, Iowa City, IA USA; 159 Johns Hopkins University, Baltimore, MD USA; 160 The University of Kansas, Lawrence, KS USA; 161 Kansas State University, Manhattan, KS USA; 162 Lawrence Livermore National Laboratory, Livermore, CA USA; 163 University of Maryland, College Park, MD USA; 164 Massachusetts Institute of Technology, Cambridge, MA USA; 165 University of Minnesota, Minneapolis, MN USA; 166 University of Mississippi, Oxford, MS USA; 167 University of Nebraska-Lincoln, Lincoln, NE USA; 168 State University of New York at Buffalo, Buffalo, NY USA; 169 Northeastern University, Boston, MA USA; 170 Northwestern University, Evanston, IL USA; 171 University of Notre Dame, Notre Dame, IN USA; 172 The Ohio State University, Columbus, OH USA; 173 Princeton University, Princeton, NJ USA; 174 University of Puerto Rico, Mayagüez, PR USA; 175 Purdue University, West Lafayette, IN USA; 176 Purdue University Northwest, Hammond, IN USA; 177 Rice University, Houston, TX USA; 178 University of Rochester, Rochester, NY USA; 179 The Rockefeller University, New York, NY USA; 180 Rutgers, The State University of New Jersey, Piscataway, NJ USA; 181 University of Tennessee, Knoxville, TN USA; 182 Texas A &M University, College Station, TX USA; 183 Texas Tech University, Lubbock, TX USA; 184 Vanderbilt University, Nashville, TN USA; 185 University of Virginia, Charlottesville, VA USA; 186 Wayne State University, Detroit, MI USA; 187 University of Wisconsin-Madison, Madison, WI USA; 188 Authors Affiliated with an Institute or an International Laboratory Covered by a Cooperation Agreement with CERN, Geneva, Switzerland; 189 Yerevan State University, Yerevan, Armenia; 190 TU Wien, Vienna, Austria; 191 Institute of Basic and Applied Sciences, Faculty of Engineering, Arab Academy for Science, Technology and Maritime Transport, Alexandria, Egypt; 192 Ghent University, Ghent, Belgium; 193 Universidade Estadual de Campinas, Campinas, Brazil; 194 Federal University of Rio Grande do Sul, Porto Alegre, Brazil; 195 UFMS, Nova Andradina, Brazil; 196 Nanjing Normal University, Nanjing, China; 197 The University of Iowa, Iowa City, IA USA; 198 University of Chinese Academy of Sciences, Beijing, China; 199 China Center of Advanced Science and Technology, Beijing, China; 200 University of Chinese Academy of Sciences, Beijing, China; 201 China Spallation Neutron Source, Dongguan, Guangdong China; 202 Henan Normal University, Xinxiang, China; 203 Université Libre de Bruxelles, Brussels, Belgium; 204 an Institute or an International Laboratory Covered by a Cooperation Agreement with CERN, Geneva, Switzerland; 205 British University in Egypt, Cairo, Egypt; 206 Cairo University, Cairo, Egypt; 207 Purdue University, West Lafayette, IN USA; 208 Université de Haute Alsace, Mulhouse, France; 209 Department of Physics, Tsinghua University, Beijing, China; 210 The University of the State of Amazonas, Manaus, Brazil; 211 Erzincan Binali Yildirim University, Erzincan, Turkey; 212 University of Hamburg, Hamburg, Germany; 213 III. Physikalisches Institut A, RWTH Aachen University, Aachen, Germany; 214 Isfahan University of Technology, Isfahan, Iran; 215 Bergische University Wuppertal (BUW), Wuppertal, Germany; 216 Brandenburg University of Technology, Cottbus, Germany; 217 Forschungszentrum Jülich, Jülich, Germany; 218 CERN, European Organization for Nuclear Research, Geneva, Switzerland; 219 Institute of Physics, University of Debrecen, Debrecen, Hungary; 220 Institute of Nuclear Research ATOMKI, Debrecen, Hungary; 221 Universitatea Babes-Bolyai-Facultatea de Fizica, Cluj-Napoca, Romania; 222 Physics Department, Faculty of Science, Assiut University, Asyût, Egypt; 223 HUN-REN Wigner Research Centre for Physics, Budapest, Hungary; 224 Punjab Agricultural University, Ludhiana, India; 225 University of Visva-Bharati, Santiniketan, India; 226 Indian Institute of Science (IISc), Bangalore, India; 227 Birla Institute of Technology, Mesra, Mesra, India; 228 IIT Bhubaneswar, Bhubaneswar, India; 229 Institute of Physics, Bhubaneswar, India; 230 University of Hyderabad, Hyderabad, India; 231 Deutsches Elektronen-Synchrotron, Hamburg, Germany; 232 Department of Physics, Isfahan University of Technology, Isfahan, Iran; 233 Sharif University of Technology, Tehran, Iran; 234 Department of Physics, University of Science and Technology of Mazandaran, Behshahr, Iran; 235 Helwan University, Cairo, Egypt; 236 Italian National Agency for New Technologies, Energy and Sustainable Economic Development, Bologna, Italy; 237 Centro Siciliano di Fisica Nucleare e di Struttura Della Materia, Catania, Italy; 238 Università degli Studi Guglielmo Marconi, Rome, Italy; 239 Scuola Superiore Meridionale, Università di Napoli ‘Federico II’, Naples, Italy; 240 Fermi National Accelerator Laboratory, Batavia, IL USA; 241 Ain Shams University, Cairo, Egypt; 242 Consiglio Nazionale delle Ricerche-Istituto Officina dei Materiali, Perugia, Italy; 243 Riga Technical University, Riga, Latvia; 244 Department of Applied Physics, Faculty of Science and Technology, Universiti Kebangsaan Malaysia, Bangi, Malaysia; 245 Consejo Nacional de Ciencia y Tecnología, Mexico City, Mexico; 246 Trincomalee Campus, Eastern University, Sri Lanka, Nilaveli, Sri Lanka; 247 Saegis Campus, Nugegoda, Sri Lanka; 248 National and Kapodistrian University of Athens, Athens, Greece; 249 Ecole Polytechnique Fédérale Lausanne, Lausanne, Switzerland; 250 Universität Zürich, Zurich, Switzerland; 251 Stefan Meyer Institute for Subatomic Physics, Vienna, Austria; 252 Laboratoire d’Annecy-le-Vieux de Physique des Particules, IN2P3-CNRS, Annecy-le-Vieux, France; 253 Near East University, Research Center of Experimental Health Science, Mersin, Turkey; 254 Konya Technical University, Konya, Turkey; 255 Izmir Bakircay University, Izmir, Turkey; 256 Adiyaman University, Adiyaman, Turkey; 257 Bozok Universitetesi Rektörlügü, Yozgat, Turkey; 258 Marmara University, Istanbul, Turkey; 259 Milli Savunma University, Istanbul, Turkey; 260 Kafkas University, Kars, Turkey; 261 Istanbul Okan University, Istanbul, Turkey; 262 Hacettepe University, Ankara, Turkey; 263 Faculty of Engineering, Istanbul University-Cerrahpasa, Istanbul, Turkey; 264 Yildiz Technical University, Istanbul, Turkey; 265 Vrije Universiteit Brussel, Brussels, Belgium; 266 School of Physics and Astronomy, University of Southampton, Southampton, UK; 267 University of Bristol, Bristol, UK; 268 IPPP Durham University, Durham, UK; 269 Faculty of Science, Monash University, Clayton, Australia; 270 Università di Torino, Turin, Italy; 271 Bethel University, St. Paul, MN USA; 272 Karamanoğlu Mehmetbey University, Karaman, Turkey; 273 California Institute of Technology, Pasadena, CA USA; 274 United States Naval Academy, Annapolis, MD USA; 275 Bingol University, Bingol, Turkey; 276 Georgian Technical University, Tbilisi, Georgia; 277 Sinop University, Sinop, Turkey; 278 Erciyes University, Kayseri, Turkey; 279 Horia Hulubei National Institute of Physics and Nuclear Engineering (IFIN-HH), Bucharest, Romania; 280 an Institute or an International Laboratory Covered by a Cooperation Agreement with CERN, Geneva, Switzerland; 281 Texas A &M University at Qatar, Doha, Qatar; 282 Kyungpook National University, Daegu, Korea; 283 Another Institute or International Laboratory Covered by a Cooperation Agreement with CERN, Geneva, Switzerland; 284 Universiteit Antwerpen, Antwerp, Belgium; 285 Yerevan Physics Institute, Yerevan, Armenia; 286 Northeastern University, Boston, MA USA; 287 Imperial College, London, UK; 288 Institute of Nuclear Physics of the Uzbekistan Academy of Sciences, Tashkent, Uzbekistan; 289 CERN, 1211 Geneva 23, Switzerland

## Abstract

A study of the anomalous couplings of the Higgs boson to vector bosons, including ${\textit{CP}}$-violation effects, has been conducted using its production and decay in the WW channel. This analysis is performed on proton–proton collision data collected with the CMS detector at the CERN LHC during 2016–2018 at a center-of-mass energy of 13 TeV, and corresponds to an integrated luminosity of 138$\,\text {fb}^{-1}$. The different-flavor dilepton $({\textrm{e}} {{\upmu }})$ final state is analyzed, with dedicated categories targeting gluon fusion, electroweak vector boson fusion, and associated production with a W or Z boson. Kinematic information from associated jets is combined using matrix element techniques to increase the sensitivity to anomalous effects at the production vertex. A simultaneous measurement of four Higgs boson couplings to electroweak vector bosons is performed in the framework of a standard model effective field theory. All measurements are consistent with the expectations for the standard model Higgs boson and constraints are set on the fractional contribution of the anomalous couplings to the Higgs boson production cross section.

## Introduction

After the discovery of the Higgs boson (H) by the ATLAS and CMS Collaborations in 2012 [1–3], the CMS [4–11] and ATLAS [12–18] experiments set constraints on the spin-parity properties of the Higgs boson and its couplings with gluons and electroweak (EW) gauge bosons, denoted here as Hgg and HVV, respectively. The Higgs boson quantum numbers are consistent with the standard model (SM) expectation $J^{PC} = 0^{++},$ but the possibility of small, anomalous couplings is not yet ruled out. In beyond-the-SM (BSM) theories, interactions with the Higgs boson may occur through several anomalous couplings, which lead to new tensor structures in the interaction terms that can be both ${\textit{CP}}$-even or ${\textit{CP}}$-odd. The ${\textit{CP}}$-odd anomalous couplings between the Higgs boson and BSM particles may generate ${\textit{CP}}$ violation in the interactions of the Higgs boson.

In this paper, we study the tensor structure of the Hgg and HVV couplings, and we search for several anomalous effects, including ${\textit{CP}}$ violation, using the different-flavor dilepton $({\textrm{e}} {{\upmu }})$ final state from $\textrm{H}\rightarrow \textrm{WW}$ decays. The Higgs boson production processes include gluon fusion (ggH), EW vector boson fusion (VBF), and associated production with a W or Z boson (VH). Higgs boson production and decay processes are sensitive to certain anomalous contributions, which can be described by higher-dimensional operators in an effective field theory (EFT) [19] that can modify the kinematic distributions of the Higgs boson decay products and the particles from associated production.

Each production process of the Higgs boson is identified using its kinematic features, and events are assigned to corresponding production categories. The matrix element likelihood approach (MELA) [20–24] is employed to construct observables that are optimal for the measurement of anomalous couplings, or EFT operators, at the production vertex. These and other decay-based variables are used to explore all kinematic features of the events, giving the analysis sensitivity to simultaneous anomalous effects at the Higgs boson production and decay vertices. Fully simulated signal samples that include anomalous couplings incorporate the detector response into the analysis.

The analysis is based on the proton–proton $(\textrm{pp})$ collision data collected at the CERN LHC from 2016 to 2018, at a center-of-mass energy of 13 TeV, corresponding to an integrated luminosity of 138$\,\text {fb}^{-1}$. This paper builds on a previous analysis conducted by the CMS Collaboration in the $\textrm{H}\rightarrow \textrm{WW}$ channel [25], which focused on measuring the Higgs boson production cross sections and coupling parameters in the so-called $\kappa$ framework [26]. We follow a formalism used in previous CMS analyses of anomalous couplings in Run 1 and Run 2 [4–11, 27, 28], focusing on the case where the Higgs boson is produced on-shell. The coupling parameters are extracted using the signal strength and the fractional contributions of the couplings to the cross section. A general study of the HVV interaction is performed with four anomalous couplings analyzed individually. Through SU(2) x U(1) symmetry considerations, the anomalous HVV couplings are reduced in number to three and analyzed simultaneously. The primary HVV coupling measurements are performed in terms of cross section fractions with additional interpretations in terms of EFT couplings included. A study of the Hgg interaction is also performed in terms of a ${\textit{CP}}$-odd anomalous coupling cross section fraction.

This paper is organized as follows. The phenomenology of anomalous couplings is discussed in Sect. 2. Section 3 gives a brief overview of the CMS apparatus. Data sets and Monte Carlo (MC) simulation samples are discussed in Sect. 4. The event reconstruction and selection are outlined in Sects. 5 and 6, respectively. Methods to estimate backgrounds are given in Sect. 7. In Sect. 8, we discuss the kinematic variables associated with Higgs boson production and decay. Sources of systematic uncertainties are presented in Sect. 9. The results are presented and discussed in Sect. 10. Finally, a summary is given in Sect. 11. Tabulated results are provided in the HEPData record for this analysis [29].

## Phenomenology

In this analysis, we investigate anomalous coupling effects in gluon fusion or electroweak Higgs boson production, as well as in its decay to WW pairs. A detailed discussion of the theoretical considerations can be found in Refs. [22, 24, 28]. The interaction of the spin-zero Higgs boson with two spin-one gauge bosons  such as WW, $\textrm{ZZ}$, ${\textrm{Z}} {{\upgamma }},$ ${{\upgamma }}{{\upgamma }},$ or gg, can be parametrized by the scattering amplitude1where  and  are the spin-one gauge boson four-momentum and polarization vectors, $m_{\textrm{V}1}$ is the pole mass of the boson,  and ${\tilde{f}}^{(i)}_{\mu \nu } = \frac{1}{2} \epsilon _{\mu \nu \rho \sigma } f^{(i),\rho \sigma }$ (with $\epsilon _{\mu \nu \rho \sigma }$ the Levi-Civita symbol),  is the scale of BSM physics, and *v* is the Higgs field vacuum expectation value.

The only leading tree-level contributions in the scattering amplitude are $a_{1}^\textrm{ZZ}\ne 0$ and $a_{1}^{\textrm{WW}} \ne 0;$ other $a_{1}$ coupling parameters $({\textrm{Z}} {{\upgamma }},$ ${{\upgamma }}{{\upgamma }},$ gg) do not contribute because the pole mass vanishes. Additional $\textrm{ZZ}$ and WW couplings are considered anomalous contributions. Anomalous terms arising in the SM via loop effects are typically small and are not yet accessible experimentally. The BSM contributions, however, could yield larger coupling parameters. Among the anomalous contributions, considerations of symmetry and gauge invariance require $\kappa _1^\textrm{ZZ} = \kappa _2^\textrm{ZZ},$ $\kappa _1^{{{\upgamma }}{{\upgamma }}} = \kappa _2^{{{\upgamma }}{{\upgamma }}} = 0,$ $\kappa _1^{\textrm{gg}} = \kappa _2^{\textrm{gg}} = 0,$ and $\kappa _1^{{\textrm{Z}} {{\upgamma }}} = 0$ [24]. The presence of ${\textit{CP}}$-odd  couplings together with any of the other couplings (all of them ${\textit{CP}}$-even), will result in ${\textit{CP}}$ violation. We reduce the number of independent parameters by assuming that $a_2^{{{\upgamma }}{{\upgamma }}},$ $a_3^{{{\upgamma }}{{\upgamma }}},$ $a_2^{{\textrm{Z}} {{\upgamma }}}$ and $a_3^{{\textrm{Z}} {{\upgamma }}}$ are constrained in direct decays of ${\textrm{H}} \rightarrow {{\upgamma }}{{\upgamma }}$ and ${\textrm{Z}} {{\upgamma }},$ therefore fixing them to be zero. The $a^{\textrm{gg}}_{2}$ term results from loop effects in the SM.

The relationship between the $\textrm{ZZ}$ and WW couplings is mostly relevant for VBF production. There are no kinematic differences between the $\textrm{ZZ}$ and WW fusion processes; therefore, it is not possible to disentangle the couplings. One possibility is to set the $\textrm{ZZ}$ and WW couplings to be equal, $a_{i} = a_{i}^\textrm{ZZ} = a_{i}^{\textrm{WW}},$ leaving four HVV anomalous couplings to be measured: $a_2,$ $a_3,$ $\kappa _1/(\varLambda _1)^2,$ and $\kappa _2^{{\textrm{Z}} {{\upgamma }}}/(\varLambda _1^{{\textrm{Z}} {{\upgamma }}})^2.$ The $a_{1}^\textrm{ZZ} = a_{1}^{\textrm{WW}}$ relationship also appears under custodial symmetry. This approach provides a general test of the Higgs boson Lagrangian tensor structure and a search for ${\textit{CP}}$ violation in HVV interactions. In an alternative approach, the SU(2) $\times$ U(1) symmetry reduces the number of independent HVV anomalous couplings to three $(a_2,$ $a_3,$ and $\kappa _1/(\varLambda _1)^2)$ through the introduction of the following coupling parameter relationships [19] :

2 $$\begin{aligned} a_1^{\textrm{WW}}&= a_1^{\textrm{ZZ}}, \end{aligned}$$

3 $$\begin{aligned} a_2^{\textrm{WW}}&= c_\text {w} ^2 a_2^{\textrm{ZZ}}, \end{aligned}$$

4 $$\begin{aligned} a_3^{\textrm{WW}}&= c_\text {w} ^2 a_3^{\textrm{ZZ}}, \end{aligned}$$

5 $$\begin{aligned} \frac{\kappa _1^{\textrm{WW}}}{(\varLambda _1^{\textrm{WW}})^2}&= \frac{1}{c_\text {w} ^2-s_\text {w} ^2} \left( \frac{\kappa _1^{\textrm{ZZ}}}{(\varLambda _1^{\textrm{ZZ}})^2} -2 s_\text {w} ^2 \frac{a_2^{\textrm{ZZ}}}{m_{\textrm{Z}} ^2}\right) , \end{aligned}$$

6 $$\begin{aligned} \frac{\kappa _2^{{\textrm{Z}} {{\upgamma }}}}{(\varLambda _1^{{\textrm{Z}} {{\upgamma }}})^2}&= \frac{ 2 s_\text {w} c_\text {w}}{c_\text {w} ^2-s_\text {w} ^2} \left( \frac{\kappa _1^{\textrm{ZZ}}}{(\varLambda _1^{\textrm{ZZ}})^2} - \frac{a_2^{\textrm{ZZ}}}{m_{\textrm{Z}} ^2}\right) , \end{aligned}$$

where $c_\text {w}$ and $s_\text {w}$ are the cosine and sine of the weak mixing angle, respectively, and $m_{\textrm{Z}}$ is the ${\textrm{Z}}$ boson mass. With this approach, there is a linear relationship between the scattering amplitude couplings and the SM EFT (SMEFT) couplings in the Higgs basis [19]:

7 $$\begin{aligned} \delta c_{\text {z}}&= \frac{1}{2} a_1^{\textrm{ZZ}} - 1, \end{aligned}$$

8 $$\begin{aligned} c_{\text {zz}}&= -\frac{2 s_\text {w} ^2 c_\text {w} ^2}{e^2} a_2^{\textrm{ZZ}}, \end{aligned}$$

9 $$\begin{aligned} \tilde{c}_{\text {zz}}&= -\frac{2 s_\text {w} ^2 c_\text {w} ^2}{e^2} a_3^{\textrm{ZZ}}, \end{aligned}$$

10 $$\begin{aligned} c_{\text {z} \Box }&= \frac{m_{\textrm{Z}} ^2 s_\text {w} ^2}{e^2} \, \frac{\kappa _1^{\textrm{ZZ}}}{(\varLambda _1^{\textrm{ZZ}})^2}, \end{aligned}$$

where *e* is the electron charge. The amplitude couplings may also be related to the SMEFT Warsaw basis [19, 30] couplings through the following translation [28, 31] :

11 $$\begin{aligned} \delta a_1^{\textrm{ZZ}}&= \frac{v^2}{\varLambda ^2} \left( 2c_{\text {H}\Box } + \frac{6e^2}{s_\text {w} ^2}c_{\text {HWB}} + \left( \frac{3c_\text {w} ^2}{2s_\text {w} ^2} -\frac{1}{2}\right) c_{\text {HD}} \right) , \end{aligned}$$

12 $$\begin{aligned} \kappa _1^{\textrm{ZZ}}&= \frac{v^2}{\varLambda ^2} \left( -\frac{2e^2}{s_\text {w} ^{2}}c_{\text {HWB}} + \left( 1-\frac{1}{2s_\text {w} ^2}\right) c_{\text {HD}} \right) , \end{aligned}$$

13 $$\begin{aligned} a_2^{\textrm{ZZ}}&= -2\frac{v^2}{\varLambda ^2} \left( s_\text {w} ^2 c_{\text {HB}} + c_\text {w} ^2 c_{\text {HW}} + s_\text {w} c_\text {w} c_{\text {HWB}} \right) , \end{aligned}$$

14 $$\begin{aligned} a_3^{\textrm{ZZ}}&= -2\frac{v^2}{\varLambda ^2} \left( s_\text {w} ^2 c_{\text {H}\tilde{\text {B}}} + c_\text {w} ^2 c_{\text {H}\tilde{\text {W}}} + s_\text {w} c_\text {w} c_{\text {H}\tilde{\text {W}}\text {B}} \right) , \end{aligned}$$

where $\varLambda$ is the UV cutoff of the theory (set to 1 TeV), and $\delta a_1^{\textrm{ZZ}}$ is a correction to the SM value of $a_1^{\textrm{ZZ}}.$ Further discussion on the EFT operators corresponding to the couplings considered here may be found in Chapter 2.2 of Ref. [19]. The assumed constraints on $a_2^{{{\upgamma }}{{\upgamma }}},$ $a_3^{{{\upgamma }}{{\upgamma }}},$ $a_2^{{\textrm{Z}} {{\upgamma }}}$ and $a_3^{{\textrm{Z}} {{\upgamma }}}$ imply that only one of the three coupling parameters $c_{\text {HW}},$ $c_{\text {HWB}},$ and $c_{\text {HB}}$ is independent; the same is also true for their ${\textit{CP}}$-odd counterparts $c_{\text {H}\tilde{\text {W}}},$ $c_{\text {H}\tilde{\text {W}}\text {B}},$ and $c_{\text {H}\tilde{\text {B}}}.$ Therefore, we have four independent HVV couplings in both the Higgs and Warsaw basis. All the EFT couplings are expected to be zero in the SM.

We thus adopt two approaches to the HVV coupling study. In Approach 1, we use the $a_{i}^\textrm{ZZ} = a_{i}^{\textrm{WW}}$ relationship and individually analyze each of the four anomalous couplings. In Approach 2, we enforce the SU(2) x U(1) relationships from Eqs. (2–6) and analyze the three independent anomalous couplings both individually and simultaneously. Approach 1 may be considered to follow the relationships from Eqs. (2–5) in the limiting case $c_\text {w} = 1.$

It is convenient to measure the fractional contribution of the anomalous couplings to the Higgs boson cross section rather than the anomalous couplings themselves. For the anomalous HVV couplings, the effective fractional cross sections $f_{ai}$ are defined as15where $\sum _{j}$ sums over all the coupling parameters considered, including $a_1,$ and $\sigma _{i}$ is the cross section for the process corresponding to $a_{i} = 1$ and $a_{j \ne i} = 0.$ Many systematic uncertainties cancel out in the ratio, and the physical range is conveniently bounded between $-1$ and $+1.$ Our primary measurements are performed in terms of cross section fractions, with additional interpretations in terms of the SMEFT Higgs and Warsaw basis couplings also included. For consistency with previous CMS measurements, the $\sigma _i$ coefficients used to define the fractional cross sections correspond to the $\textrm{gg}\rightarrow {\textrm{H}} \rightarrow \textrm{VV} \rightarrow 2{\textrm{e}} 2{{\upmu }}$ process [28]. The numerical values are given in Table 1 as calculated using the JHUGen simulation [20–23]. Two sets of values are shown corresponding to the different coupling relationships adopted in Approach 1 and 2.

It has been shown that the angular correlations of the associated jets in the ggH + 2 jets process are sensitive to anomalous Hgg coupling effects at the production vertex [32]. The quark-quark initiated process, $\textrm{qq} \rightarrow \textrm{qqH},$ corresponds to the gluon scattering topology sensitive to anomalous effects. For the anomalous Hgg coupling, the effective fractional cross section can be defined as16The $\sigma _3^\text {gg}$ and $\sigma _{2}^{\textrm{gg}}$ cross sections correspond to $a_3^{\textrm{gg}} = 1, a_2^{\textrm{gg}} = 0$ and $a_2^{\textrm{gg}} = 1, a_3^{\textrm{gg}} = 0,$ respectively, and are equal. With this analysis it is not possible to distinguish the top quark, bottom quark, and heavy BSM particle contributions in the gluon fusion loop. As such, the Hgg coupling is treated as an effective coupling with heavy degrees of freedom integrated out.

**Table 1:** The cross sections $(\sigma _i)$ of the anomalous contributions $(a_i)$ relative to the SM value $(\sigma _1)$ used to define the fractional cross sections $f_{ai}$ for the Approach 1 and 2 coupling relationships. For the $\kappa _{1}$ and $\kappa _{2}^{{\textrm{Z}} {{\upgamma }}}$ couplings, the numerical values $\varLambda _1 = \varLambda _1^{{\textrm{Z}} {{\upgamma }}} = 100\,\text {Ge}\hspace{-.08em}\text {V}$ are chosen to keep all coefficients of similar order of magnitude

$f_{ai}$	$a_i$	Approach 1 $\sigma _{i}/\sigma _{1}$	Approach 2 $\sigma _{i}/\sigma _{1}$
$f_{a2}$	$a_2$	0.361	6.376
$f_{a3}$	$a_3$	0.153	0.153
$f_{\varLambda 1}$	$\kappa _1$	0.682	5.241
$f_{\varLambda 1}^{{\textrm{Z}} {{\upgamma }}}$	$\kappa _2^{{\textrm{Z}} {{\upgamma }}}$	1.746	${-}$

## The CMS detector

The CMS apparatus [33] is a multipurpose, nearly hermetic detector, designed to identify electrons, muons, photons, and (charged and neutral) hadrons [34–37]. A global reconstruction “particle-flow” (PF) algorithm [38] combines the information provided by the all-silicon inner tracker and by the crystal electromagnetic and brass-scintillator hadron calorimeters, operating inside a 3.8$\,\text {T}$ superconducting solenoid, with data from gas-ionization muon detectors interleaved with the solenoid return yoke, to build $\uptau$leptons, jets, missing transverse momentum, and other physics objects [39–41].

Events of interest are selected using a two-tiered trigger system [42, 43]. The first level (L1), composed of custom hardware processors, uses information from the calorimeters and muon detectors to select events at a rate of around 100$\,\text {kHz}$ within a fixed latency of about 4$\,\upmu \text {s}$ [42]. The second level, known as the high-level trigger (HLT), consists of a farm of processors running a version of the full event reconstruction software optimized for fast processing, and reduces the event rate to around 1$\,\text {kHz}$ before data storage [43]. A more detailed description of the CMS detector, together with a definition of the coordinate system and kinematic variables, can be found in Ref. [33].

## Data sets and simulation

The data sets included in this analysis were recorded with the CMS detector in 2016, 2017, and 2018, and correspond to integrated luminosities of 36.3, 41.5, and 59.7$\,\text {fb}^{-1}$, respectively [44–46]. The collision events must fulfill HLT selection criteria that require the presence of one or two leptons satisfying isolation and identification requirements. For the 2016 data set, the single-electron trigger has a transverse momentum ($p_{\textrm{T}}$) threshold of 25$\,\text {Ge}\hspace{-.08em}\text {V}$ for electrons with pseudorapidity $\left| {\eta }\right| < 2.1$ and 27$\,\text {Ge}\hspace{-.08em}\text {V}$ for $2.1< \left| {\eta }\right| < 2.5,$ whereas the single-muon trigger has a $p_{\textrm{T}}$ threshold of 24$\,\text {Ge}\hspace{-.08em}\text {V}$ for $\left| {\eta }\right| < 2.4.$ For the 2017 (2018) data set, the $p_{\textrm{T}}$ threshold is 35 (32)$\,\text {Ge}\hspace{-.08em}\text {V}$ for the single-electron trigger (covering $\left| {\eta }\right| < 2.5)$ and 27 (24)$\,\text {Ge}\hspace{-.08em}\text {V}$ for the single-muon trigger $(\left| {\eta }\right| <2.4)$. The dilepton ${\textrm{e}} {{\upmu }}$ trigger has $p_{\textrm{T}}$ thresholds of 23 and 12$\,\text {Ge}\hspace{-.08em}\text {V}$ for the leading and subleading leptons, respectively, with the same coverage in pseudorapidity for electrons and muons as above. During the first part of data taking in 2016, a lower $p_{\textrm{T}}$ threshold of 8$\,\text {Ge}\hspace{-.08em}\text {V}$ for the subleading muon was used.

Monte Carlo event generators are used to model the signal and background processes. For each process, three independent sets of simulated events, corresponding to the three years of data taking, are used. This approach includes year-dependent effects in the CMS detector, data taking, and event reconstruction. All simulated events corresponding to a given data set share the same set of parton distribution functions (PDFs), underlying event (UE) tune, and parton shower (PS) configuration. The PDF sets used are NNPDF 3.0 [47, 48] for 2016 and NNPDF 3.1 [49] for 2017 and 2018. The CUETP8M1 [50] tune is used to describe the UE in 2016 simulations, whereas the CP5 [51] tune is adopted in 2017 and 2018 simulated events. The MC samples are interfaced with pythia 8.226 [52] in 2016, and 8.230 in 2017 and 2018, for the modeling of UE, PS, and hadronization. Standard Model Higgs boson production through ggH, VBF, and VH is simulated at next-to-leading order (NLO) accuracy in quantum chromodynamics (QCD), including finite quark mass effects, using powheg v2 [53–59]. The minlo hvj [58] extension of powheg v2 is used for the simulation of $\textrm{WH}$ and quark-induced $\textrm{ZH}$ production, providing NLO accuracy for the $\textrm{VH}+0$- and 1-jet processes. For ggH production, the simulated events are weighted to match the NNLOPS [60, 61] prediction in the hadronic jet multiplicity ($N_{\text {jet}}$) and Higgs boson $p_{\textrm{T}}$ distributions. The weighting is based on $p_{\textrm{T}}$ and $N_{\text {jet}}$ as computed in the simplified template cross section scheme 1.0 [62]. The minlo hjj [63] generator, which provides NLO accuracy for $N_{\text {jet}} \ge 2,$ is also used for ggH production. The associated production processes with top quarks ($\textrm{t}{\bar{\textrm{t}}}\textrm{H}$) and bottom quarks (${\textrm{b}{\bar{\textrm{b}}}\textrm{H}}$) are simulated with powheg v2 and MadGraph 5_amc@nlo v2.2.2 [64], respectively, and have a negligible contribution in the analysis phase space. All SM Higgs boson samples are normalized to the cross sections recommended in Ref. [19]. The Higgs boson mass in the event generation is assumed to be 125$\,\text {Ge}\hspace{-.08em}\text {V}$, while a value of 125.38$\,\text {Ge}\hspace{-.08em}\text {V}$ [65] is used for the calculation of cross sections and branching fractions. The decay to a pair of W bosons and subsequently to leptons or hadrons is performed using the JHUGen v5.2.5 generator in 2016, and v7.1.4 in 2017 and 2018, for ggH, VBF, and quark-induced $\textrm{ZH}$ samples. The Higgs boson and W boson decays are performed using pythia 8.212 for the other signal simulations.

The ggH, VBF, and VH Higgs boson events with HVV anomalous couplings are generated with JHUGen at LO accuracy. With respect to the $\kappa _2^{{\textrm{Z}} {{\upgamma }}}/(\varLambda _1^{{\textrm{Z}} {{\upgamma }}})^2$ coupling parameter discussed in Sect. 2, the sign convention of the photon field is determined by the sign in front of the gauge fields in the covariant derivative. In this analysis, we define the covariant derivative $D_\mu = \partial _\mu -\textrm{i}e \sigma ^i W_\mu ^i/(2s_w) + \textrm{i}e B_\mu /(2 c_w)$ following the convention in JHUGen [31]. The JHUGen and powheg SM Higgs boson simulations were compared after parton showering and no significant differences in the distributions of kinematic observables were found. We adopt the JHUGen simulation to describe the kinematic features in all production modes with HVV anomalous couplings. The expected yields are scaled to match the SM theoretical predictions [19] for inclusive cross sections and the powheg SM prediction of relative event yields in the event categorization based on associated particles. Simulation of the ggH + 2 jets process with Hgg anomalous couplings is done using minlo X0jj [66] at NLO in QCD. A large number of signal samples with various anomalous couplings were generated. The MELA package [20–24] contains a library of matrix elements from JHUGen for different Higgs boson signal hypotheses. Matrix elements from different coupling signal hypotheses, but with the same production mechanism, are used to reweight the generated signal events. This procedure is used in the construction of the predictions for the different coupling components and their interference, allowing us to cover all points in the signal model phase space with sufficient statistical precision.

Background events are produced using several simulations. The quark-initiated nonresonant WW process is simulated with powheg v2 [67] at NLO accuracy for inclusive production. A reweighting is performed to match the diboson $p_{\textrm{T}}$ spectrum computed at NNLO+NNLL QCD accuracy [68, 69]. The mcfm v7.0 [70–72] generator is used to simulate gluon-induced WW production at LO accuracy, with the normalization chosen to match the NLO cross section [73]. Nonresonant EW production of WW pairs with two additional jets is simulated at LO accuracy with MadGraph 5_amc@nlo v2.4.2 using the MLM matching and merging scheme [74]. Top quark pair production ($\textrm{t}{\bar{\textrm{t}}}$) and single top quark processes, including $\textrm{tW}$, s- and t-channel contributions, are simulated with powheg v2 [75–77]. A reweighting of the top quark and antiquark $p_{\textrm{T}}$ spectrum at parton level is performed for the $\textrm{t}{\bar{\textrm{t}}}$simulation in order to match the NNLO and next-to-next-to-leading logarithm (NNLL) QCD predictions, including also the NLO EW contribution [78].

The Drell–Yan (DY) production of a charged-lepton pair is simulated with MadGraph 5_amc@nlo v2.4.2 at NLO accuracy with up to two additional partons, using the FxFx matching and merging scheme [79]. Production of a W boson associated with an initial state radiation photon (${\textrm{W}} {}{{\upgamma }}$) is simulated with MadGraph 5_amc@nlo v2.4.2 at NLO accuracy with up to 1 additional parton, using the FxFx jet merging. Diboson processes containing at least one Z boson or a virtual photon $({{\upgamma }}^{*})$ with a mass as low as 100$\,\text {Me}\hspace{-.08em}\text {V}$ are generated with powheg v2 [67] at NLO accuracy. Production of a W boson in association with a ${{\upgamma }}^{*}$ (${\textrm{W}} {}{{{\upgamma }}}^{*}$) for masses below 100$\,\text {Me}\hspace{-.08em}\text {V}$ is simulated by pythia 8.212 in the parton showering of ${\textrm{W}} {}{{\upgamma }}$ events. Triboson processes with inclusive decays are also simulated at NLO accuracy with MadGraph 5_amc@nlo v2.4.2.

For all processes, the detector response is simulated using a detailed description of the CMS detector, based on the Geant4 toolkit [80]. The distribution of additional $\textrm{pp}$ interactions within the same or nearby bunch crossings (pileup) in the simulation is reweighted to match that observed in data. The efficiency of the trigger system is evaluated in data on a per lepton basis using dilepton events consistent with the Z boson decay. The overall efficiencies of the trigger selections used in the analysis are obtained as the average of the per-lepton efficiencies weighted by their probability. The resulting efficiencies are applied directly on simulated events.

## Event reconstruction

The identification and measurement of the properties of individual particles (PF candidates) in an event is achieved in the PF algorithm by combining information from various subdetectors. Electrons are identified and their momenta are measured in the pseudorapidity interval $\left| {\eta }\right| < 2.5$ by combining tracks in the silicon tracker with spatially compatible energy deposits in the electromagnetic calorimeter. Muons are identified and their momenta are measured in the pseudorapidity range $\left| {\eta }\right| < 2.4$ by matching tracks in the muon system and the silicon tracker. For better rejection of nonprompt leptons, increasing the sensitivity of the analysis, leptons are required to be isolated and well reconstructed using a set of criteria based on the quality of the track reconstruction, shape of calorimetric deposits, and energy flux in the vicinity of the particle’s trajectory [34, 35]. In addition, a selection based on a dedicated multivariate analysis (MVA) tagger developed for the CMS $\textrm{t}{\bar{\textrm{t}}}\textrm{H}$ analysis [81] is added in all channels for muon candidates.

Multiple $\textrm{pp}$ interaction vertices are identified from tracking information by use of the adaptive vertex fitting algorithm [82]. The primary $\textrm{pp}$ interaction vertex is taken to be the vertex corresponding to the hardest scattering in the event, evaluated using tracking information alone, as described in Section 9.4.1 of Ref. [83]. Leptons are required to be associated to the primary vertex using transverse and longitudinal impact parameter criteria [34, 35].

Hadronic jets are clustered from PF candidates using the infrared- and collinear-safe anti-$k_{\textrm{T}}$ algorithm with distance parameters of 0.4 (AK4) and 0.8 (AK8). The jet momentum is determined as the vectorial sum of all particle momenta in the jet. The AK8 jets considered are required to be reconstructed within the silicon tracker acceptance $(\left| {\eta }\right| < 2.4)$, whereas AK4 jets are reconstructed in the range $\left| {\eta }\right| < 4.7.$ For AK4 jets, contamination from pileup is suppressed using charged-hadron subtraction which removes charged PF candidates originating from vertices other than the primary interaction vertex. The residual contribution from neutral particles originating from pileup vertices is removed by means of an event-by-event jet-area-based correction to the jet four-momentum [84]. For AK8 jets, the pileup-per-particle identification algorithm (PUPPI) [85] is used to mitigate the effect of pileup at the reconstructed-particle level, making use of local shape information, event pileup properties, and tracking information. Additional selection criteria are applied to remove jets potentially dominated by instrumental effects or reconstruction failures [84].

The AK8 jets are used to reconstruct hadronic Vboson decays in a single merged jet when the decay products are highly collimated. This approach targets boosted W or Z bosons originating from the VH production mode. Such Lorentz-boosted Vdecays are identified using the ratio of the 2- to 1-subjettiness [86], $\tau _{2}/\tau _{1}$, and the groomed jet mass $m_{\text {J}}.$ The groomed mass is calculated after applying a modified mass drop algorithm [87, 88], known as the soft-drop algorithm [89], with parameters $\beta = 0,$ $z_\text {cut} = 0.1,$ and $R_0 = 0.8.$ The algorithm also identifies two hard subjets within the AK8 jet.

We refer to the identification of jets likely originating from bottom quarks as b tagging [90, 91]. For each AK4 jet in the event, a score is calculated through a multivariate combination of different jet properties, making use of boosted decision trees and deep neural networks. A jet is considered b-tagged if its associated score exceeds a threshold, tuned to achieve a certain tagging efficiency as measured in $\textrm{t}{\bar{\textrm{t}}}$events. The chosen working point corresponds to about 90% efficiency for bottom quark jets and to a mistagging rate of about 10% for light-flavor or gluon jets and of about 50% for charm quark jets.

The missing transverse momentum vector ${\vec p}_{\textrm{T}}^{\hspace{1.66656pt}\text {miss}}$ is computed as the negative vector sum of the transverse momenta of all the PF candidates in an event, and its magnitude is denoted as $p_{\textrm{T}} ^\text {miss}$ [41]. The PUPPI algorithm is applied to reduce the pileup dependence of the ${\vec p}_{\textrm{T}}^{\hspace{1.66656pt}\text {miss}}$ observable by computing the ${\vec p}_{\textrm{T}}^{\hspace{1.66656pt}\text {miss}}$ from the PF candidates weighted by their probability to originate from the primary interaction vertex [41].

## Event selection

The analysis is performed using $\textrm{H}\rightarrow \textrm{WW}$ candidate events in the ${\textrm{e}} {{\upmu }}$ final state. For an event to be selected, the transverse momenta of the leading lepton $p_{\textrm{T}} ^{\ell 1}$ and the subleading lepton $p_{\textrm{T}} ^{\ell 2}$ must be greater than 25 and 13$\,\text {Ge}\hspace{-.08em}\text {V}$, respectively. The $p_{\textrm{T}} ^{\ell 2}$ threshold in the case of a muon is lowered to 10$\,\text {Ge}\hspace{-.08em}\text {V}$ for the 2016 data set because of the lower threshold in the corresponding HLT algorithm. Events containing additional leptons with $p_{\textrm{T}} > 10\,\text {Ge}\hspace{-.08em}\text {V}$ are discarded. The dilepton system is required to have an invariant mass $m_{\ell \ell }$ greater than 12$\,\text {Ge}\hspace{-.08em}\text {V}$ and transverse momentum $p_{\textrm{T}} ^{\ell \ell }$ above 30$\,\text {Ge}\hspace{-.08em}\text {V}$. A requirement on the missing transverse momentum of $p_{\textrm{T}} ^\text {miss} > 20\,\text {Ge}\hspace{-.08em}\text {V}$ is implemented. We define transverse mass discriminating variables $m_{\textrm{T}} ^{{\textrm{H}}}$ and $m_{\textrm{T}} ^{\ell 2}$ as

17 $$\begin{aligned}{} & {} m_{\textrm{T}} ^{{\textrm{H}}} = \sqrt{2p^{\ell \ell }_{\textrm{T}} p_{\textrm{T}} ^\text {miss} [1-\cos {\varDelta \varPhi (\vec {p}_{\text {T}}^{\ell \ell },\vec {p}_{\text {T}}^{\text {miss}})}]}, \end{aligned}$$

18 $$\begin{aligned}{} & {} m_{\textrm{T}} ^{\ell 2} = \sqrt{2p_{\textrm{T}} ^{\ell 2} p_{\textrm{T}} ^\text {miss} [1-\cos {\varDelta \varPhi (\vec {p}_{\text {T}}^{\ell 2},\vec {p}_{\text {T}}^{\text {miss}})}]}, \end{aligned}$$

and select events with $m_{\textrm{T}} ^{{\textrm{H}}} > 60\,\text {Ge}\hspace{-.08em}\text {V}$ and $m_{\textrm{T}} ^{\ell 2} > 30\,\text {Ge}\hspace{-.08em}\text {V}.$ The $m_{\textrm{T}} ^{{\textrm{H}}}$ requirement suppresses the $\text {DY}\rightarrow \uptau {}\uptau$ background process and avoids overlap with the $H\rightarrow \uptau \uptau$ analysis [11]. To ensure orthogonality with a future off-shell $\textrm{H}\rightarrow \textrm{WW}$ analysis we require $m_{\textrm{T}} ^{{\textrm{H}}} < 125\,\text {Ge}\hspace{-.08em}\text {V}.$ In addition, the region $76.2< m_{\ell \ell } < 106.2\,\text {Ge}\hspace{-.08em}\text {V}$ is excluded to avoid overlap with the off-shell $\textrm{H}\rightarrow \textrm{ZZ} \rightarrow 2\ell 2{{\upnu }}$ analysis [10]. These requirements will simplify a future combination of Higgs boson decay final states. Finally, events with any b-tagged jets with $p_{\textrm{T}} > 20\,\text {Ge}\hspace{-.08em}\text {V}$ are vetoed. These base selection criteria are summarized in Table 2.

**Table 2:** Summary of the base selection criteria

Variable	Selection
Number of leptons	2 $({\textrm{e}} {{\upmu }}$ of opposite charge)
$p_{\textrm{T}} ^{\ell 1}$	$>25$ $\,\text {Ge}\hspace{-.08em}\text {V}$
$p_{\textrm{T}} ^{\ell 2}$	$>13$ $\,\text {Ge}\hspace{-.08em}\text {V}$ (10$\,\text {Ge}\hspace{-.08em}\text {V}$ for 2016 data)
$m_{\ell \ell }$	12–76.2$\,\text {Ge}\hspace{-.08em}\text {V}$ or >106.2$\,\text {Ge}\hspace{-.08em}\text {V}$
$p_{\textrm{T}} ^{\ell \ell }$	$>30$ $\,\text {Ge}\hspace{-.08em}\text {V}$
$p_{\textrm{T}} ^\text {miss}$	$>20$ $\,\text {Ge}\hspace{-.08em}\text {V}$
$m_{\textrm{T}} ^{\ell 2}$	$>30$ $\,\text {Ge}\hspace{-.08em}\text {V}$
$m_{\textrm{T}} ^{{\textrm{H}}}$	60–125$\,\text {Ge}\hspace{-.08em}\text {V}$
$N_{\text {jet}} ({\textrm{b}} \text {jets})$	0

For the HVV coupling analysis, exclusive selection criteria, which are based on the associated jet activity in the event, are applied that target the ggH, VBF, and VH production processes. The AK4 (AK8) jets considered are required to have $p_{\textrm{T}} > 30~(200)\,\text {Ge}\hspace{-.08em}\text {V}.$ In the ggH channel, zero or one AK4 jet is required in the event. For the VBF and Resolved VH channels, we require two AK4 jets with dijet masses of $m_{\text {jj}} > 120\,\text {Ge}\hspace{-.08em}\text {V}$ and $60< m_{\text {jj}} < 120\,\text {Ge}\hspace{-.08em}\text {V},$ respectively. The Boosted VH channel requires the presence of a V-tagged AK8 jet (Vjet); such jets have a groomed mass in the region $65< m_{\text {J}} < 105\,\text {Ge}\hspace{-.08em}\text {V}$ and satisfy the requirement $\tau _{2}/\tau _{1} < 0.4.$ In the other channels, a Vjet veto is implemented to ensure orthogonality. These production channels for the HVV coupling study are summarized in Table 3.

**Table 3:** Summary of the ggH, VBF, and VH production channels used for the HVV coupling study

Variable	ggH	VBF	Resolved VH	Boosted VH
$N_{\text {jet}} (\textrm{V}\text {jets})$	0	0	0	$>0$
$N_{\text {jet}} (\text {AK4 jets})$	0 and 1	2	2	${-}$
$m_{\text {jj}}$	${-}$	$>120$ $\,\text {Ge}\hspace{-.08em}\text {V}$	60–120$\,\text {Ge}\hspace{-.08em}\text {V}$	${-}$

As the production vertex of the ggH + 2 jets process is sensitive to anomalous Hgg coupling effects, we use a 2-jet ggH channel that follows the VBF selection described above for the Hgg coupling analysis. The $\mathrm HWW$decay vertex is not sensitive to anomalous Hgg effects, and so decay-based variables are not studied in this channel. This permits a relatively tight selection of $m_{\ell \ell } < 55\,\text {Ge}\hspace{-.08em}\text {V}$ which is beneficial for background suppression. The 0- and 1-jet ggH channels are also included to constrain the ggH signal strength. All channels included for the Hgg coupling study are summarized in Table 4.

**Table 4:** Summary of ggH channel selections used for the Hgg coupling study

Variable	ggH	2-jet ggH
$N_{\text {jet}}$ (AK4 jets)	0 and 1	2
$m_{\text {jj}}$	${-}$	$>120$ $\,\text {Ge}\hspace{-.08em}\text {V}$
$m_{\ell \ell }$	${-}$	$<55$ $\,\text {Ge}\hspace{-.08em}\text {V}$

Control regions (CRs) are defined using the base selection criteria together with a set of alternative requirements summarized in Table 5. They are used to validate the background description and to estimate the number of background events in the signal region (SR). A dedicated $\uptau {}\uptau$ CR targets events from the DY process ${\textrm{Z}} \rightarrow \uptau {}\uptau$ with $\uptau$ leptons decaying leptonically to produce the ${\textrm{e}} {{\upmu }}$ final state. Also a top quark CR is defined to enhance events with one or more top quarks decaying to a W boson and bottom quark. Splitting events according to the number of associated jets, separate $\uptau {}\uptau$ and top quark CRs are defined for the 0-, 1- and 2-jet SRs. An additional CR with an enhanced contribution from the nonresonant WW background is used in the 2-jet SR. All CRs are used in the final data fit to constrain the DY, top quark, and WW background normalizations.

**Table 5:** Summary of the $\uptau {}\uptau ,$ top quark, and WW control region requirements

Variable	$\uptau {}\uptau$	Top quark	WW
$m_{\ell \ell }$	40–80$\,\text {Ge}\hspace{-.08em}\text {V}$	$>50$ $\,\text {Ge}\hspace{-.08em}\text {V}$	$>106.2$ $\,\text {Ge}\hspace{-.08em}\text {V}$
$m_{\textrm{T}} ^{{\textrm{H}}}$	$<60$ $\,\text {Ge}\hspace{-.08em}\text {V}$	${-}$	60–125$\,\text {Ge}\hspace{-.08em}\text {V}$
$m_{\textrm{T}} ^{\ell 2}$	${-}$	$>30$ $\,\text {Ge}\hspace{-.08em}\text {V}$	$>30$ $\,\text {Ge}\hspace{-.08em}\text {V}$
	0	$>0$	0

Additional $\uptau {}\uptau ,$ top quark, and WWCRs are defined requiring a Vjet. These CRs are used to validate the background description in the Boosted VH channel. However, they generally do not have a sufficient number of events to significantly constrain the background normalizations in the final fit to the data. As such, we rely on the 2-jet CRs to determine the normalizations to be used in the Boosted VH channel. Agreement between data and the background prediction in the Vjet CRs is observed when using normalizations determined in the 2-jet CRs.

## Background estimation

The nonprompt-lepton backgrounds originating from leptonic decays of heavy quarks, hadrons misidentified as leptons, and electrons from photon conversions are suppressed by identification and isolation requirements imposed on electrons and muons. In this analysis, the nonprompt-lepton background primarily originates from ${\textrm{W}} +$jets events and is estimated from data, as described in detail in Ref. [92]. The procedure involves measuring the rate at which a nonprompt lepton passing a loose selection further passes a tight selection (misidentification rate) and the corresponding rate for a prompt lepton to pass this selection (prompt rate). The misidentification rate is measured in a data sample enriched in multijet events, whereas the prompt rate is measured using a tag-and-probe method [93] in a data sample enriched in DY events. The nonprompt-lepton background estimation is validated with data in a CR enriched with ${\textrm{W}} +$jets events, in which a pair of same-sign leptons is required.

The backgrounds from top quark processes and nonresonant WW production are estimated using a combination of MC simulations and the dedicated CRs described in the previous section. The normalisations of these backgrounds are left as free parameters in the fit, keeping different parameters for each jet multiplicity region. The top quark background normalization is measured from the observed data in the top quark enriched CRs. A separate normalization parameter is included for the quark-induced and gluon-induced WW backgrounds. For the 2-jet regions, the WW enriched CR is used to constrain the WW background normalisation parameters. In the 0- and 1-jet channels, these parameters are constrained directly in the signal regions, which span the high $m_{\ell \ell }$ phase space enriched in WW events.

The $\text {DY}\rightarrow \uptau {}\uptau$ background process is estimated with a data-embedding technique [94]. As for the top quark and WW backgrounds, the DY normalization is left unconstrained in the data fit. The $\text {DY}\rightarrow \uptau {}\uptau$ enriched CR described in Sect. 6 is used to constrain the free normalization parameters in the 0-, 1-, 2-jet regions. The data-embedded samples cover the events that pass the ${\textrm{e}} {{\upmu }}$ triggers, which represent the vast majority of the selected events. The remaining $\text {DY}\rightarrow \uptau {}\uptau$ events, which enter the analysis through the single-lepton triggers $({\approx }5\%$ of the total), are estimated using MC simulation.

**Fig. 1:**
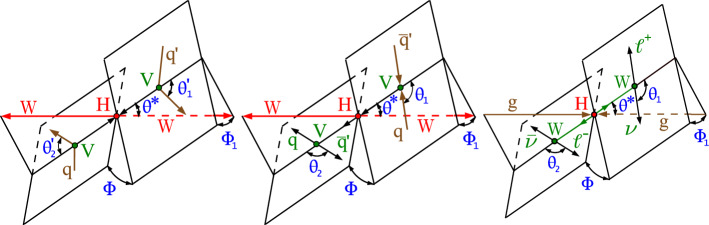
Topologies of the Higgs boson production and decay for vector boson fusion ${\textrm{q}} {{\textrm{q}} ^\prime }\rightarrow {\textrm{q}} {{\textrm{q}} ^\prime } {\textrm{H}}$ (left), ${\textrm{q}} \bar{{\textrm{q}}}^\prime \rightarrow \textrm{VH}$ (center), and gluon fusion with decay $\textrm{gg} \rightarrow {\textrm{H}} \rightarrow 2 \ell 2\nu$ (right). For the electroweak production topologies, the intermediate vector bosons and their decays are shown in green and the ${\textrm{H}} \rightarrow \textrm{WW}$ decay is marked in red. For the $\textrm{gg} \rightarrow {\textrm{H}} \rightarrow 2 \ell 2\nu$ topology, the ${\textrm{W}}$ boson leptonic decays are shown in green. In all cases, the incoming particles are depicted in brown and the angles characterizing kinematic distributions are marked in blue. Five angles fully characterize the orientation of the production and decay chain and are defined in the suitable rest frames

The $\textrm{WZ}$ and ${\textrm{W}} {}{{{\upgamma }}}^{*}$ background contributions are simulated as described in Sect. 4, and a data-to-simulation scale factor is derived in a three-lepton CR, as described in Ref. [92]. The contribution of the ${\textrm{W}} {}{{\upgamma }}$ process may also be a background because of photon conversions in the detector material. This process is estimated using MC simulation and validated using data in a CR requiring events with a leading $\upmu$ and a trailing e with same sign and a separation in $\varDelta R=\sqrt{\smash [b]{\varDelta \phi ^2 + \varDelta \eta ^2}}$ (where $\phi$ is the azimuthal angle in radians) smaller than 0.5. Triple vector boson production is a minor background in all channels and is estimated using MC simulation.

## Observables and kinematic discriminants

In this paper, we search for anomalous HVV and Hgg coupling effects by studying:

1. the two quark jets from VBF and VH production (HVV coupling);
2. the $\textrm{H}\rightarrow \textrm{WW}$ decay products (HVV coupling); and
3. the two quark jets from ggH + 2 jets production (Hgg coupling).

The VBF, VH, and ggH production and decay topologies relevant for the HVV coupling are illustrated in Fig. 1.

When combined with the momentum transfer of the vector bosons, the five angles illustrated for VBF/VH production provide complete kinematic information for production and decay of the Higgs boson. The illustration for Higgs boson production via ggH in association with two jets is identical to the VBF diagram, except for replacing the intermediate vector bosons by gluons. Full production kinematic information is extracted for VBF, VH, and ggH + 2 jets candidate events using discriminants built from the matrix element calculations of the MELA package. The MELA approach is designed to reduce the number of observables to a minimum, while retaining all essential information. To form the production-based MELA kinematic discriminants, we use jets to reconstruct the four-momentum of the associated production particles. The presence of two neutrinos in the final state means it is not possible to reconstruct the four-momentum of all the Higgs boson decay products. Therefore, decay-based kinematic discriminants built from matrix elements are not used in this analysis. Instead, we rely on kinematic variables related to the measured final state of the Higgs boson decay. The strategies used for each of the topologies listed above are now discussed in more detail.

### Kinematic features of two quark jets in VBF and VH channels

Kinematic distributions of associated particles in VBF and VH production are sensitive to the anomalous HVV couplings of the Higgs boson.

As illustrated in Fig. 1, a set of seven observables can be defined for the VBF and VH production topologies: $\varOmega = \{\theta _{1}^{(\prime )}, \theta _{2}^{(\prime )}, \theta ^*, \varPhi , \varPhi _1, q_1^{2}, q_2^{2} \},$ with $q_1^2$ and $q_2^2$ the squared four-momenta of the vector bosons [22]. Three types of discriminants are defined using the full kinematic description characterized by $\varOmega .$ The first type of discriminant is designed to separate signal and background Higgs boson production processes:

19 $$\begin{aligned} {\mathcal {D}}_{\text {sig}} = \frac{{\mathcal {P}}_{\text {sig}}(\varOmega ) }{{\mathcal {P}}_{\text {sig}}(\varOmega ) +{\mathcal {P}}_{\text {bkg}}(\varOmega )}, \end{aligned}$$

where the probability density ${\mathcal {P}}$ for a specific process is calculated from the matrix elements provided by the MELA package. The second type of discriminant separates the anomalous coupling BSM process from that of the SM:

20 $$\begin{aligned} {\mathcal {D}}_{\text {BSM}} = \frac{{\mathcal {P}}_{\text {BSM}}(\varOmega ) }{{\mathcal {P}}_{\text {BSM}}(\varOmega ) + {\mathcal {P}}_{\text {SM}}(\varOmega )}. \end{aligned}$$

Throughout this document the generic BSM label is generally replaced by the specific anomalous coupling state targeted. For the $a_{3}$ ${\textit{CP}}$-odd and $a_{2}$ ${\textit{CP}}$-even coupling parameters, we use, respectively, ${\mathcal {D}}_{{0-}}$ and ${\mathcal {D}}_{0+},$ whereas for the $\varLambda _1$ coupling parameters we use ${\mathcal {D}}_{\varLambda _1}$ and ${\mathcal {D}}_{\varLambda _1}^{{\textrm{Z}} \gamma }.$ The third type of discriminant isolates the interference contribution:

21 $$\begin{aligned} {\mathcal {D}}_{\text {int}} = \frac{ {\mathcal {P}}_{\text {SM-BSM}}^{\text {int}}(\varOmega )}{{\mathcal {P}}_{\text {SM}}(\varOmega ) +{\mathcal {P}}_{\text {BSM}}(\varOmega )}, \end{aligned}$$

where ${\mathcal {P}}_{\text {SM-BSM}}^{\text {int}}$ is the interference part of the probability distribution for a process with a mixture of the SM and BSM contributions. The ${\textit{CP}}$ label is generally used for the $a_3$ coupling parameter, as the BSM signal in this case is a pseudoscalar and the interference discriminant is a ${\textit{CP}}$-sensitive observable. The ${\mathcal {P}}$ values are normalized to give the same integrated cross sections in the relevant phase space of each process. Such normalization leads to a balanced distribution of events in the range between 0 and 1 for ${\mathcal {D}}_{\text {sig}}$ and ${\mathcal {D}}_{\text {BSM}},$ and between $-1$ and $+1$ for ${\mathcal {D}}_{\text {int}}.$

**Fig. 2:**
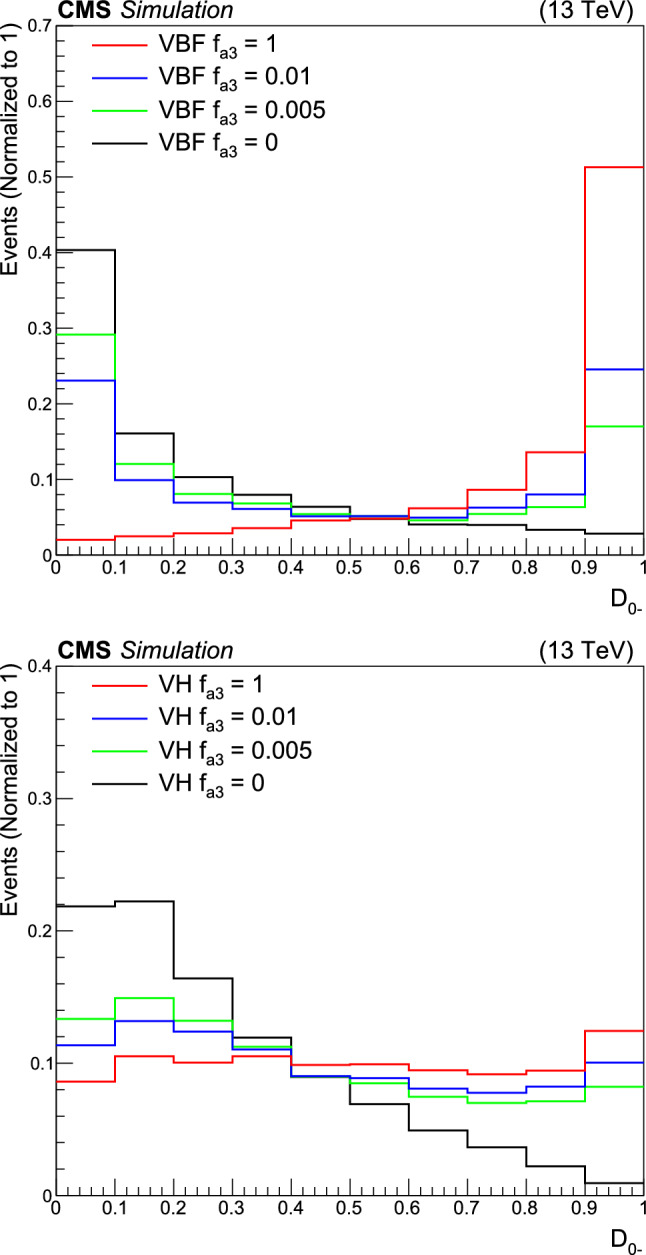
The $${\mathcal {D}}_{{0-}}$$ discriminant in the VBF (upper) and Resolved VH (lower) production channels for a number of VBF (upper) and VH (lower) signal hypotheses. Pure $$a_1$$ $$(f_{a3} = 0)$$ and $$a_3$$ $$(f_{a3} = 1)$$ HVV signal hypotheses are shown along with two mixed coupling hypotheses $$(f_{a3} = 0.005$$ and $$f_{a3} = 0.01)$$. All distributions are normalized to unity

The selected events are split into three main production channels: VBF, Resolved VH, and Boosted VH. In the first two channels, the four-momenta of the two AK4 jets assigned as the associated particles are used in the MELA probability calculation. For the Boosted VH category, we use the four-momentum of the two subjets of the V-tagged AK8 jet. An estimate of the Higgs boson four-momentum is also required for the probability calculation. This can not be measured directly since the final state contains two neutrinos. As such, we construct a proxy Higgs boson four-momentum in the following manner. The $p_{\text {x}}$ and $p_{\text {y}}$ of the dineutrino system are estimated from the ${\vec p}_{\textrm{T}}^{\hspace{1.66656pt}\text {miss}}$ in a given event. The corresponding $p_{\text {z}}$ is then set to equal that of the dilepton system, which is based on the observed correlation between these variables at the generator level for simulated signals. Finally, the mass of the dineutrino system is set equal to the mean value of the generator-level dineutrino mass. The resulting four-momentum can then be combined with that of the measured dilepton system to create a proxy Higgs boson four-momentum. We note that the MELA probability calculation for the production vertices is largely based on the kinematic features of the associated particles, so the reconstruction of the proxy Higgs boson has a relatively small effect on the final discriminants. As an illustrative example of the MELA based discriminants used in this analysis, Fig. 2 shows the ${\mathcal {D}}_{{0-}}$ discriminant in the VBF and Resolved VH production channels for a number of different signal hypotheses. The discriminants are designed to target the dominant signal production process in a given channel.

In the VBF channel, a ${\mathcal {D}}_{\text {VBF}}$ discriminant is constructed, following Eq. (19), where ${\mathcal {P}}_{\text {sig}}$ corresponds to the probability for the VBF production hypothesis, and ${\mathcal {P}}_{\text {bkg}}$ corresponds to that of gluon fusion production in association with two jets. The discriminant is also suitable for separating SM backgrounds from the VBF signal process. In the Resolved and Boosted VH channels, the corresponding discriminants do not give a significant level of separation with respect to ggH production or SM backgrounds. This is due to the relatively tight selection criteria, which limit the phase space to VH-like events. Hence, these discriminants are not included in the VH channels.

The ${\mathcal {D}}_{{\textit{CP}}}$ discriminant is sensitive to the sign of the interference between the ${\textit{CP}}$-even SM and ${\textit{CP}}$-odd BSM states. An asymmetry between the number of events detected with positive and negative ${\mathcal {D}}_{{\textit{CP}}}$ values is expected for mixed ${\textit{CP}}$ states. Therefore, a forward-backward categorization (forward defined as ${\mathcal {D}}_{{\textit{CP}}} > 0$ and backward as ${\mathcal {D}}_{{\textit{CP}}} < 0)$ is used to analyze the ${\textit{CP}}$-odd couplings. Similarly, ${\mathcal {D}}_{\text {int}}$ gives sensitivity to the sign of the interference between the SM and $a_2$ HVV BSM states. A forward-backward ${\mathcal {D}}_{\text {int}}$ categorization is also included. The value of ${\mathcal {D}}_{\text {int}}$ used to define the categories is chosen to symmetrize the SM Higgs boson expectation. In the case of the $\varLambda _1$ measurements, the interference discriminants were shown to be highly correlated with the ${\mathcal {D}}_{\text {BSM}}$ discriminants and so are not considered.

We now discuss the categorization and construction of the final multidimensional discriminants used for the two HVV coupling approaches defined in Sect. 2. The binning of the final discriminants was optimized to ensure sufficient statistical precision in the predictions of all bins, while retaining the kinematic information required to discriminate between the SM and anomalous coupling signal hypotheses.

#### VBF/ VH analysis strategy for Approach 1

In Approach 1, each of the four anomalous HVV coupling parameters $(a_2,$ $a_3,$ $\kappa _1/(\varLambda _1)^2,$ and $\kappa _2^{{\textrm{Z}} {{\upgamma }}}/(\varLambda _1^{{\textrm{Z}} {{\upgamma }}})^2)$ are analyzed separately. For this purpose, we construct a multidimensional discriminant for each of the four anomalous couplings in the VBF, Resolved VH, and Boosted VH channels.

In the VBF channel, we use two bins of the production discriminant ${\mathcal {D}}_{\text {VBF}},$ corresponding to low and high purity, using a bin boundary of 0.75. The $m_{\ell \ell }$ variable, which is sensitive to anomalous effects at the $\textrm{H}\rightarrow \textrm{WW}$ decay vertex, is included with two bins in the range 12–76.2$\,\text {Ge}\hspace{-.08em}\text {V}$. A bin boundary of 45$\,\text {Ge}\hspace{-.08em}\text {V}$ is chosen based on the expected signal shape changes induced by anomalous effects. Finally, one of the ${\mathcal {D}}_{\text {BSM}}$ discriminants is included with ten equally sized bins. Depending on the anomalous coupling under study this discriminant may be ${\mathcal {D}}_{0+},$ ${\mathcal {D}}_{{0-}},$ ${\mathcal {D}}_{\varLambda _1}$ or ${\mathcal {D}}_{\varLambda _1}^{{\textrm{Z}} \gamma }.$

For the VH channels, the $m_{\ell \ell }$ and ${\mathcal {D}}_{\text {BSM}}$ observables are used to build 2D kinematic discriminants. The $m_{\ell \ell }$ bins are the same as for the VBF channel. In the Resolved VH channel, we use four ${\mathcal {D}}_{\text {BSM}}$ bins of equal size. For the Boosted VH case, three variable bins with boundaries of 0.6 and 0.8 are used, a large first bin is chosen because relatively little signal is expected at low values of ${\mathcal {D}}_{\text {BSM}}.$ A distinct multidimensional discriminant is constructed for each anomalous coupling hypothesis in the VH channels.

**Fig. 3:**
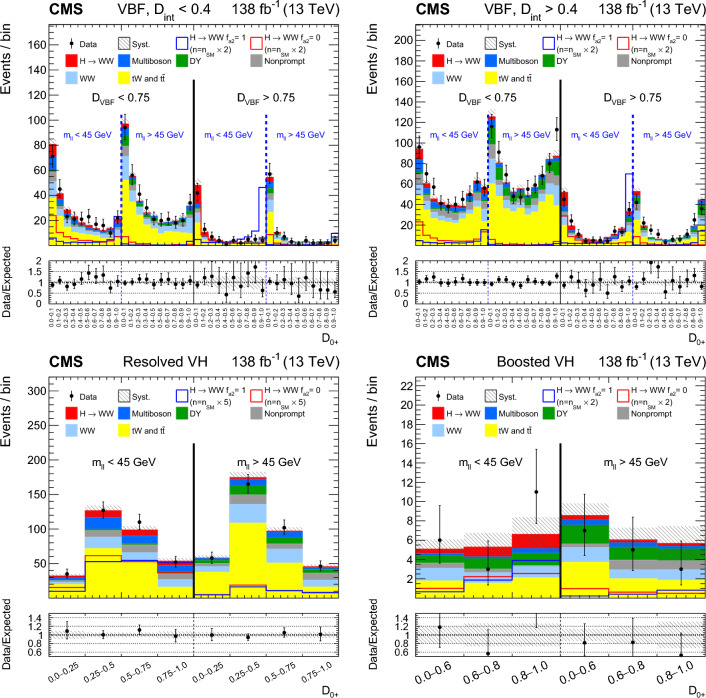
Observed and predicted distributions after fitting the data for $[{\mathcal {D}}_{\text {VBF}}, m_{\ell \ell }, {\mathcal {D}}_{0+}]$ in the VBF channel (upper), and for $[m_{\ell \ell }, {\mathcal {D}}_{0+}]$ in the Resolved VH (lower left) and Boosted VH (lower right) channels. For the VBF channel, the ${\mathcal {D}}_{\text {int}} < 0.4$ (left) and ${\mathcal {D}}_{\text {int}} > 0.4$ (right) categories are shown. The predicted Higgs boson signal is shown stacked on top of the background distributions. For the fit, the $a_{1}$ and $a_2$ HVV coupling contributions are included. The corresponding pure $a_{1}$ $(f_{a2} = 0)$ and $a_2$ $(f_{a2} = 1)$ signal hypotheses are also shown superimposed, their yields correspond to the predicted number of SM signal events scaled by an arbitrary factor to improve visibility. The uncertainty band corresponds to the total systematic uncertainty. The lower panel in each figure shows the ratio of the number of events observed to the total prediction

**Fig. 4:**
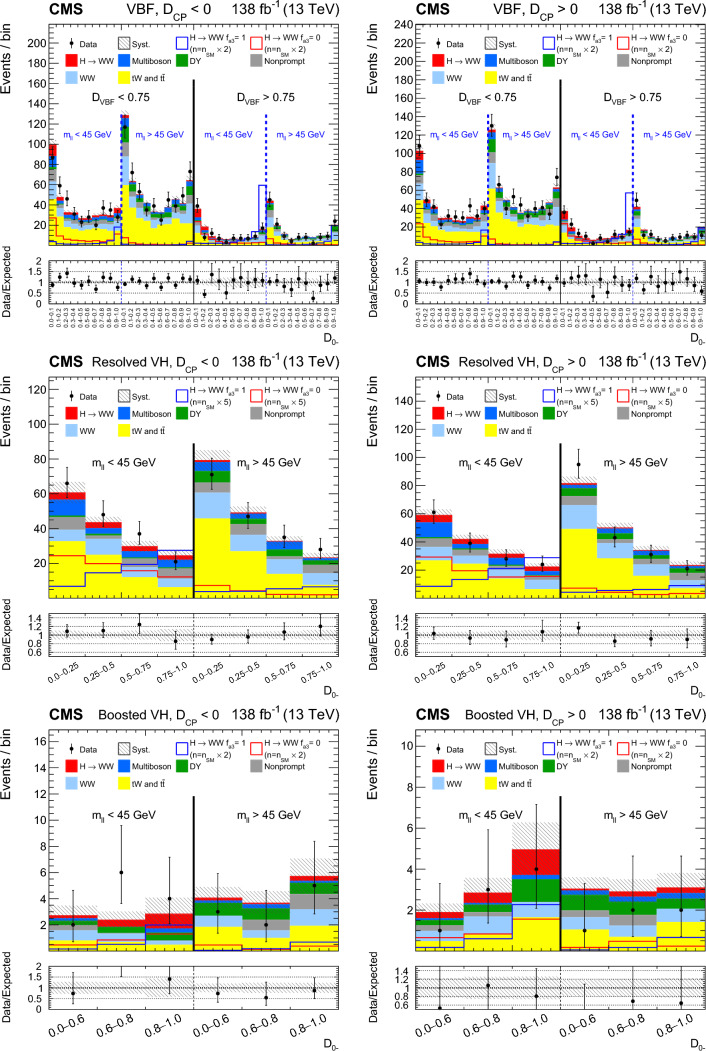
Observed and predicted distributions after fitting the data for $[{\mathcal {D}}_{\text {VBF}}, m_{\ell \ell }, {\mathcal {D}}_{{0-}}]$ in the VBF channel (upper), and for $[m_{\ell \ell }, {\mathcal {D}}_{{0-}}]$ in the Resolved VH (middle) and Boosted VH (lower) channels. For each channel, the ${\mathcal {D}}_{{\textit{CP}}} < 0$ (left) and ${\mathcal {D}}_{{\textit{CP}}} > 0$ (right) categories are shown. For the fit, the $a_{1}$ and $a_3$ HVV coupling contributions are included. More details are given in the caption of Fig. 3

**Fig. 5:**
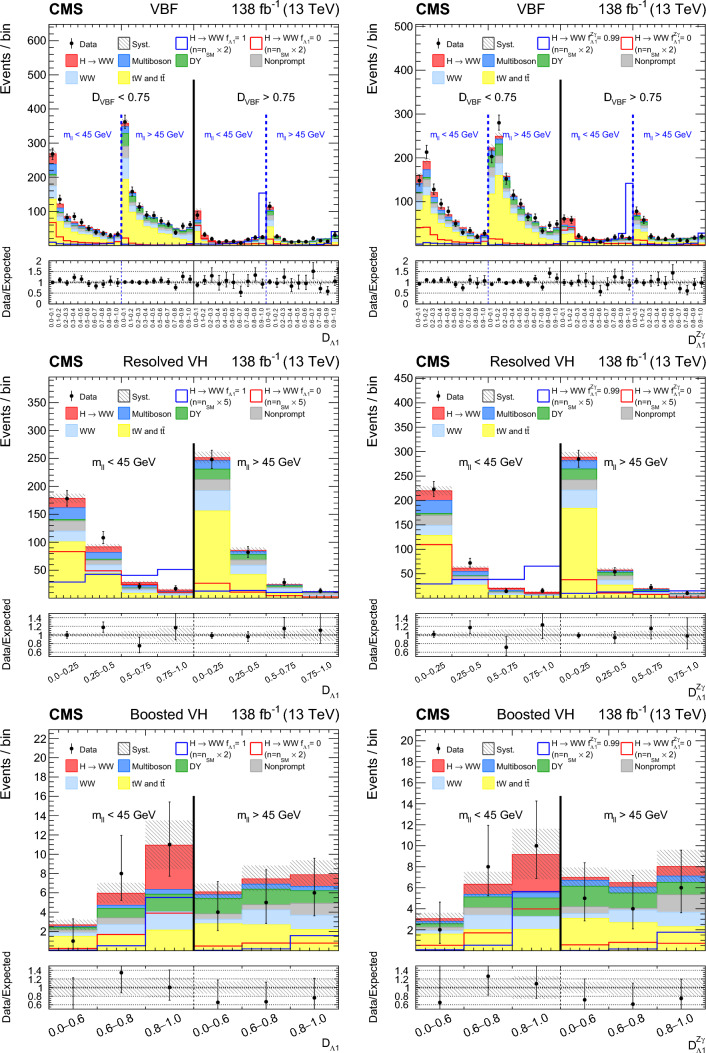
Observed and predicted distributions after fitting the data for $[{\mathcal {D}}_{\text {VBF}}, m_{\ell \ell }, {\mathcal {D}}_{\varLambda 1}]$ (upper left) and $[{\mathcal {D}}_{\text {VBF}}, m_{\ell \ell }, {\mathcal {D}}_{\varLambda 1}^{{\textrm{Z}} \gamma }]$ (upper right) in the VBF channel, and for $[m_{\ell \ell }, {\mathcal {D}}_{\varLambda 1}]$ (left) and $[m_{\ell \ell }, {\mathcal {D}}_{\varLambda 1}^{{\textrm{Z}} \gamma }]$ (right) in the Resolved VH (middle) and Boosted VH (lower) channels. For the fits, the $a_{1}$ and $\kappa _1/(\varLambda _1)^2$ (left) or $a_{1}$ and $\kappa _2^{{\textrm{Z}} {{\upgamma }}}/(\varLambda _1^{{\textrm{Z}} {{\upgamma }}})^2$ (right) HVV coupling contributions are included. More details are given in the caption of Fig. 3

For the $a_{3}$ coupling parameter, a forward-backward categorization of events based on ${\mathcal {D}}_{{\textit{CP}}}$ is implemented. In the case of the $a_{2}$ coupling parameter, ${\mathcal {D}}_{\text {int}}$ is largely correlated with ${\mathcal {D}}_{0+}$ in the VH channels. Therefore, a forward-backward ${\mathcal {D}}_{\text {int}}$ categorization is implemented only in the VBF channel. Figures 3, 4 and 5 show the discriminants used in the final fit to the data for the $a_{2},$ $a_{3},$ $\kappa _1/(\varLambda _1)^2,$ and $\kappa _2^{{\textrm{Z}} {{\upgamma }}}/(\varLambda _1^{{\textrm{Z}} {{\upgamma }}})^2$ Approach 1 coupling studies in the VBF and VH channels. A summary of the observables used in the HVV Approach 1 analysis may be found in Table 6.

#### VBF/ VH analysis strategy for Approach 2

In Approach 2, we use one categorization strategy and build one multidimensional discriminant in each channel to target all the HVV coupling parameters $(a_2,$ $a_3,$ $\kappa _1/(\varLambda _1)^2)$ simultaneously. In the VBF channel, the ${\mathcal {D}}_{{\textit{CP}}}$ and ${\mathcal {D}}_{\text {int}}$ discriminants are used to create four interference categories. Both ${\mathcal {D}}_{\text {VBF}}$ and $m_{\ell \ell }$ are used as for Approach 1. All three ${\mathcal {D}}_{\text {BSM}}$ discriminants that target the $a_{2}, a_{3}$ and $\kappa _1/(\varLambda _1)^2$ coupling parameters are included. However, the number of bins we implement is limited by the number of simulated events. Also the ${\mathcal {D}}_{\text {BSM}}$ discriminants are significantly correlated and so have similar performance for all couplings. Therefore, we use the ${\textit{CP}}$-odd discriminant ${\mathcal {D}}_{{0-}}$ and just one of the $C\!P$-even discriminants, ${\mathcal {D}}_{0+},$ both with three bins and bin boundaries of 0.1 and 0.9. A dedicated rebinning strategy is applied to the $[{\mathcal {D}}_{{0-}}, {\mathcal {D}}_{0+}]$ distribution merging bins dominated by the SM Higgs boson prediction or with low precision in the background prediction. In the VH channels, just two categories using ${\mathcal {D}}_{{\textit{CP}}}$ are defined and the discriminant is built using $m_{\ell \ell }$ as for Approach 1. Again, both ${\mathcal {D}}_{{0-}}$ and ${\mathcal {D}}_{0+}$ are chosen for the final discriminant. For the Resolved VH channel, we use three bins with boundaries of 0.25 and 0.75, whereas for the Boosted VH case we use two bins with a boundary of 0.8. The same rebinning strategy described for the VBF channel is applied to both Resolved and Boosted VH multidimensional discriminants. Table 6 includes a summary of the observables used in the HVV Approach 2 analysis.

### Kinematic features of $\textrm{H}\rightarrow \textrm{WW}$ decay products in 0- and 1-jet ggH channels

**Fig. 6:**
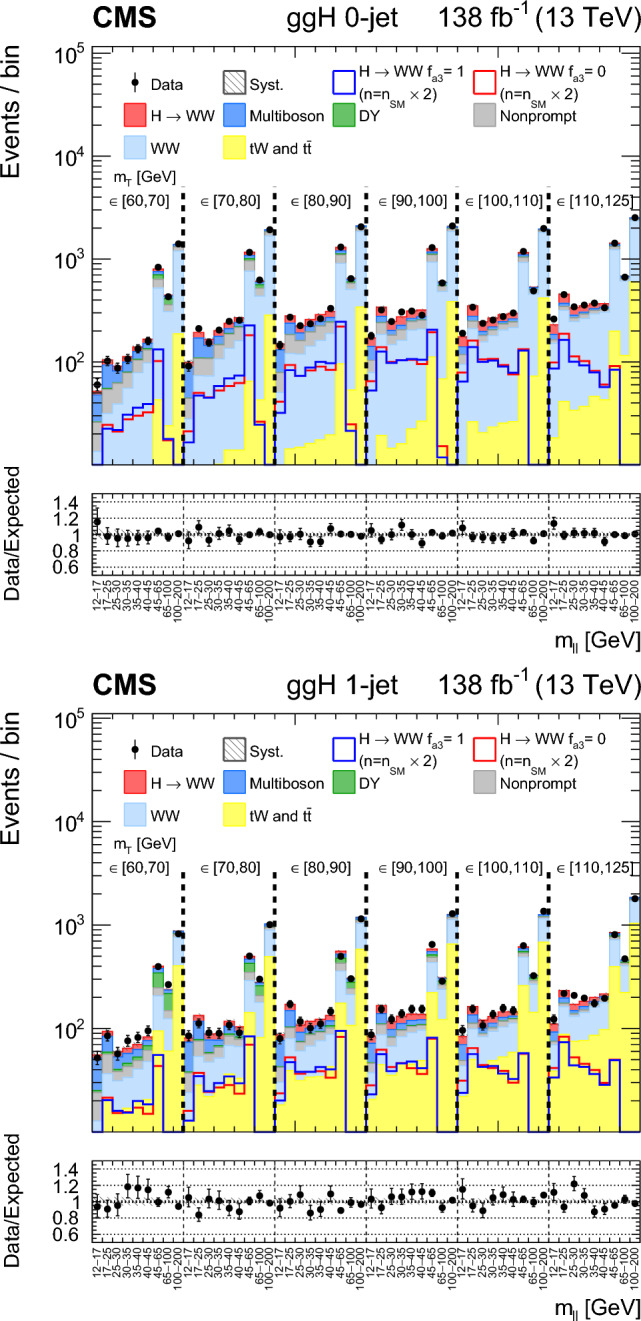
Observed and predicted distributions after fitting the data for $[m_{\textrm{T}}, m_{\ell \ell } ]$ in the 0- (upper) and 1-jet (lower) ggH channels. For the fit, the $a_{1}$ and $a_3$ HVV coupling contributions are included. More details are given in the caption of Fig. 3

Similar to the SM $\textrm{H}\rightarrow \textrm{WW}$ analysis [25], we use $m_{\ell \ell }$ and $m_{\textrm{T}}$ to build 2D discriminants in the 0- and 1-jet ggH channels. The distributions have nine bins for $m_{\ell \ell }$ in the range 12–200$\,\text {Ge}\hspace{-.08em}\text {V}$ and six bins for $m_{\textrm{T}}$ in the range 60–125$\,\text {Ge}\hspace{-.08em}\text {V}$. The bin widths vary and are optimized to achieve good separation between the SM Higgs boson signal and backgrounds, as well as between the different anomalous coupling signal hypotheses. In particular, a finer binning with respect to the SM $\textrm{H}\rightarrow \textrm{WW}$ analysis is implemented in regions where anomalous effects are most significant. Figure 6 shows the $[m_{\textrm{T}}, m_{\ell \ell } ]$ distributions in the 0- and 1-jet ggH channels. The same $[m_{\textrm{T}}, m_{\ell \ell } ]$ discriminant is used to study all HVV anomalous couplings for both Approach 1 and 2.

### Kinematic features of two quark jets in 2-jet ggH channel

**Fig. 7:**
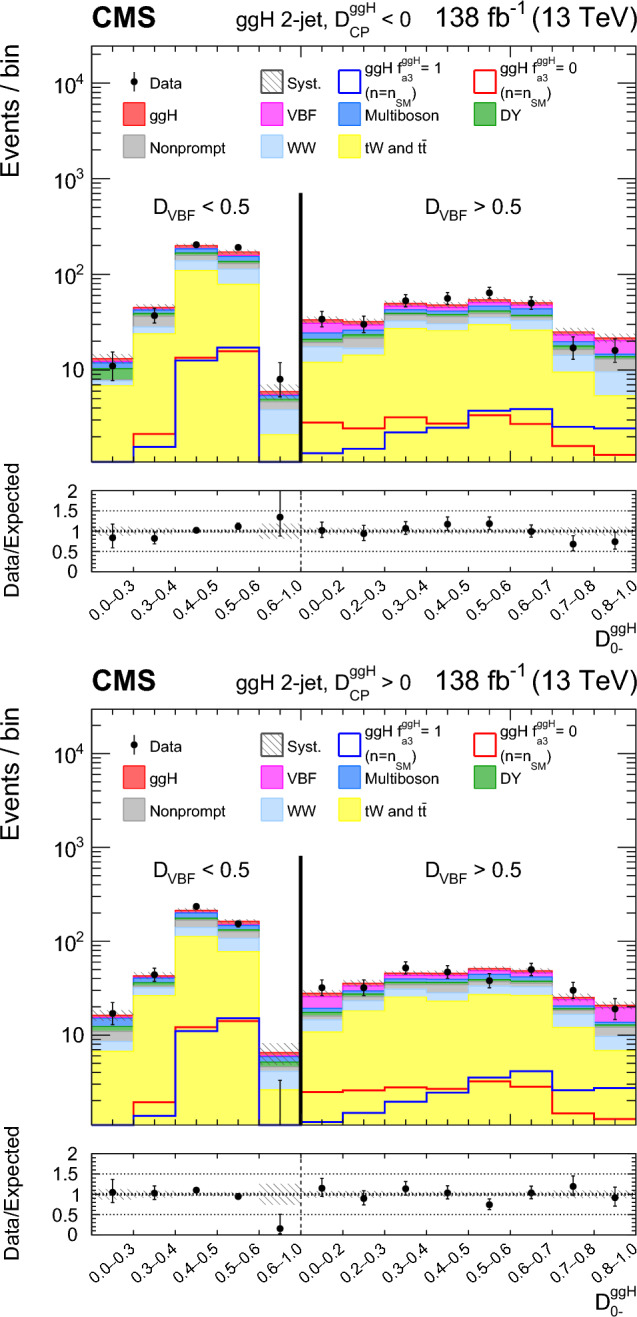
Observed and predicted distributions after fitting the data for $[{\mathcal {D}}_{\text {VBF}},$ ${\mathcal {D}}^{\textrm{ggH}}_{{0-}}]$ in the 2-jet ggH channel. Both the ${\mathcal {D}}^{\textrm{ggH}}_{\text {CP}} < 0$ (upper) and ${\mathcal {D}}^{\textrm{ggH}}_{\text {CP}} > 0$ (lower) categories are shown. In this case, the VBF and ggH signals are shown separately. For the fit, the $a_2^{\textrm{gg}}$ and $a_3^{\textrm{gg}}$ coupling contributions are included. The corresponding pure $a_2^{\textrm{gg}}$ $(f_{a3}^{\textrm{ggH}} = 0)$ and $a_3^{\textrm{gg}}$ $(f_{a3}^{\textrm{ggH}} = 1)$ signal hypotheses are also shown superimposed, their yields correspond to the predicted number of SM signal events. More details are given in the caption of Fig. 3

For the Hgg coupling, we adopt a similar approach to the VBF $C\!P$ study, where the ${\textit{CP}}$-odd $a_{3}$ HVV coupling parameter is included. In this case, the optimal observables are ${\mathcal {D}}^{\textrm{ggH}}_{{0-}}$ and ${\mathcal {D}}^{\textrm{ggH}}_{{\textit{CP}}},$ targeting the ${\textit{CP}}$-odd $a_{3}$ Hgg coupling parameter. A forward-backward categorization is implemented using ${\mathcal {D}}^{\textrm{ggH}}_{{\textit{CP}}},$ and the ${\mathcal {D}}_{\text {VBF}}$ and ${\mathcal {D}}^{\textrm{ggH}}_{{0-}}$ observables are used to build 2D discriminants. The $m_{\ell \ell }$ variable is not considered in this case because it is not sensitive to anomalous Hgg effects. For ${\mathcal {D}}_{\text {VBF}},$ the bin boundary is relaxed to 0.5 to ensure sufficient ggH events are accepted in the more VBF-like bin. For ${\mathcal {D}}_{{0-}},$ eight (five) bins are used in the more (less) VBF-like bin with larger bin sizes at the extremes of the distribution to ensure sufficient precision in the background and signal predictions. The 0- and 1-jet channels discussed previously are also included in this study to constrain the ggH signal strength. The $[{\mathcal {D}}_{\text {VBF}},$ ${\mathcal {D}}^{\textrm{ggH}}_{{0-}}]$ distributions used to analyze the Hgg $a_{3}$ anomalous coupling in the 2-jet ggH channel are shown in Fig. 7. A summary of the observables used in the Hgg analysis is given in Table 6.

**Table 6:** The kinematic observables used for the interference based categorization and for the final discriminants used in the fits to data to study the HVV and Hgg couplings. For each of the anomalous HVV couplings in Approach 1, we have a dedicated analysis in the VBF and VH channels. In Approach 2, we use one analysis to target all anomalous HVV couplings simultaneously

Analysis	Channel	Categorization	Final discriminant
HVV	VBF $(a_{2})$	${\mathcal {D}}_{\text {int}}$	$[{\mathcal {D}}_{\text {VBF}},$ $m_{\ell \ell },$ ${\mathcal {D}}_{0+}]$
Approach 1	VBF $(a_{3})$	${\mathcal {D}}_{CP}$	$[{\mathcal {D}}_{\text {VBF}},$ $m_{\ell \ell },$ ${\mathcal {D}}_{{0-}}]$
	VBF $(\kappa _1)$	${-}$	$[{\mathcal {D}}_{\text {VBF}},$ $m_{\ell \ell },$ ${\mathcal {D}}_{\varLambda 1}]$
	VBF $(\kappa _2^{{\textrm{Z}} {{\upgamma }}})$	${-}$	$[{\mathcal {D}}_{\text {VBF}},$ $m_{\ell \ell },$ ${\mathcal {D}}_{\varLambda _1}^{{\textrm{Z}} \gamma }]$
	VH $(a_{2})$	${-}$	$[m_{\ell \ell },$ ${\mathcal {D}}_{0+}]$
	VH $(a_{3})$	${\mathcal {D}}_{CP}$	$[m_{\ell \ell },$ ${\mathcal {D}}_{{0-}}]$
	VH $(\kappa _1)$	${-}$	$[m_{\ell \ell },$ ${\mathcal {D}}_{\varLambda 1}]$
	VH $(\kappa _2^{{\textrm{Z}} {{\upgamma }}})$	${-}$	$[m_{\ell \ell },$ ${\mathcal {D}}_{\varLambda _1}^{{\textrm{Z}} \gamma }]$
	0- and 1-jet ggH	${-}$	$[m_{\textrm{T}},$ $m_{\ell \ell } ]$
HVV	VBF	${\mathcal {D}}_{CP},$ ${\mathcal {D}}_{\text {int}}$	$[{\mathcal {D}}_{\text {VBF}},$ $m_{\ell \ell },$ ${\mathcal {D}}_{{0-}},$ ${\mathcal {D}}_{0+}]$
Approach 2	VH	${\mathcal {D}}_{CP}$	$[m_{\ell \ell },$ ${\mathcal {D}}_{{0-}},$ ${\mathcal {D}}_{0+}]$
	0- and 1-jet ggH	${-}$	$[m_{\textrm{T}},$ $m_{\ell \ell } ]$
Hgg	2-jet ggH	${\mathcal {D}}^{\textrm{ggH}}_{CP}$	$[{\mathcal {D}}_{\text {VBF}},$ ${\mathcal {D}}^{\textrm{ggH}}_{{0-}}]$
	0- and 1-jet ggH	${-}$	$[m_{\textrm{T}},$ $m_{\ell \ell } ]$

## Systematic uncertainties

The signal extraction is performed using binned templates to describe the various signal and background processes. Systematic uncertainties that change the normalization or shape of the templates are included. All the uncertainties are modeled as nuisance parameters that are profiled in the maximum likelihood fit described in Sect. 10. The systematic uncertainties arise from both experimental or theoretical sources.

### Experimental uncertainties

The following experimental systematic uncertainties are included in the final fit to data:

- The total uncertainty associated with the measurement of the integrated luminosity for 2016, 2017, and 2018 is $1.2\%$ [44], $2.3\%$ [45], and $2.5\%$ [46], respectively. This uncertainty is partially correlated among the three data sets, resulting in an overall uncertainty of 1.6%.
- The systematic uncertainty in the trigger efficiency is determined by varying the tag lepton selection criteria and the Z boson mass window used in the tag-and-probe method. It affects both the normalization and the shape of the signal and background distributions, and is kept uncorrelated among data sets. The total normalization uncertainty is less than 1%.
- The tag-and-probe method is also used to determine the lepton identification and isolation efficiency. Corrections are applied to account for any discrepancy in the efficiencies measured in data and simulation. The corresponding systematic uncertainty is about 1% for electrons and 2% for muons.
- The uncertainties in the determination of the lepton momentum scale mainly arise from the limited data sample used for their estimation. The impact on the normalization of the signal and background templates ranges between 0.6–1.0% for the electron momentum scale and is about 0.2% for the muon momentum scale. They are treated as uncorrelated among the three data-taking years.
- The jet energy scale uncertainty is modeled by implementing eleven independent nuisance parameters corresponding to different jet energy correction sources, six of which are correlated among the three data sets. Their effects vary in the range of 1–10%, mainly depending on the jet multiplicity in the analysis phase space. Another source of uncertainty arises from the jet energy resolution smearing applied to simulated samples to match the $p_{\textrm{T}}$ resolution measured in data. The effect varies in a range of 1–5%, depending on the jet multiplicity and is uncorrelated among the data sets. These uncertainties are included for both AK4 and AK8 jets. In addition, the $m_{\text {J}}$ scale and resolution, and Vtagging corrections with their corresponding uncertainties are included for V-tagged AK8 jets. These variables are calibrated in a top quark–antiquark sample enriched in hadronically decaying W bosons [95].
- The effects of the unclustered energy scale, jet energy scale, and lepton $p_{\textrm{T}}$ scales are included for the calculation of the missing transverse momentum. The resulting normalization systematic uncertainty is 1–10% and is treated as uncorrelated among the years.
- Both the normalization and shape of the signal and background templates are affected by the jet pileup identification uncertainty. The effect is below 1%.
- The uncertainty associated with the b tagging efficiency is modeled by seventeen nuisance parameters out of which five are of a theoretical origin and are correlated among the three data sets. The remaining set of four parameters per data set are treated as uncorrelated as they arise from the statistical accuracy of the efficiency measurement [90].
- Estimation of the nonprompt-lepton background is affected by the limited size of the data sets used for the misidentification rate measurements. It is also affected by the difference in the flavor composition of jets misidentified as leptons between the misidentification rate measurement region (enriched in multijet events) and the signal phase space. The effects on the nonprompt-lepton background estimation range between a few percent to about 10% depending on the SR and are treated as nuisance parameters uncorrelated between electrons and muons and among the three data sets. A normalization uncertainty of 30% [92] is assigned to fully cover for any discrepancies with respect to data in a ${\textrm{W}} +$jets CR and is treated as uncorrelated among data sets.
- The statistical uncertainties due to the limited number of simulated events are also included for all bins of the background distributions used to extract the results [96].

### Theoretical uncertainties

Multiple theoretical uncertainties are considered and are correlated among data sets, unless stated otherwise:

- The uncertainties related to the choice of PDF and $\alpha _\textrm{S}$ have a minor effect on the shape of the distributions. Therefore, only normalization effects related to the event acceptance and to the cross section are included. However, these uncertainties are not considered for the backgrounds that have their normalization constrained through data in dedicated CRs. For the Higgs boson signal processes, these uncertainties are calculated by the LHC Higgs cross section working group [19].
- The theoretical uncertainties arising from missing higher-order corrections in the cross section calculations are also included. Background simulations are reweighted to the alternative scenarios corresponding to renormalization $\mu _{\text {R}}$ and factorization $\mu _{\text {F}}$ scales varied by factors 0.5 or 2 and the envelopes of the varied templates are taken as the one standard deviations. For background processes that have their normalization constrained through data in dedicated CRs, we consider only the shape effect of the uncertainties coming from the missing higher-order corrections. The WWnonresonant background has the uncertainties derived by varying $\mu _{\text {R}},$ $\mu _{\text {F}},$ and the resummation scale. For the ggH and VBF signal processes, the effects of the missing higher-order corrections on the overall cross section are decoupled into multiple sources according to the recipes described in Ref. [19].
- The uncertainty due to the pileup modeling was included for the main simulated background processes (DY, WW, top quark) as well as the ggH and VBF signals. The effect is determined by varying the total inelastic $\textrm{pp}$ cross section (69.2$\,\text {mb}$ [97, 98]) within the assigned 5% uncertainty.
- The PS modeling mainly affects the jet multiplicity, causing migration of events between categories that results in template shape changes. Associated uncertainties are evaluated by reweighting events with varied PS weights computed with pythia 8.212. The effect on the signal strength is found to be below 1%.
- Uncertainties associated with UE modeling are evaluated by varying the UE tune parameters used in the MC sample generation. Systematic uncertainties are correlated between the 2017 and 2018 data sets since they share the same UE tunes, whereas for 2016 the uncertainty is considered uncorrelated. The UE uncertainty has a minimal effect on the template shapes and affects the normalization by about 1.5%.
- A 15% uncertainty is applied to the relative fraction of the gg-induced component in nonresonant WW production [99]. The relative fraction between single top quark and $\textrm{t}{\bar{\textrm{t}}}$processes is assigned a systematic uncertainty of 8% [100]. Additional process-specific (DY, ) uncertainties, related to corrections to account for possible discrepancies between data and simulation, are assigned and are correlated among data sets.

## Results

**Fig. 8:**
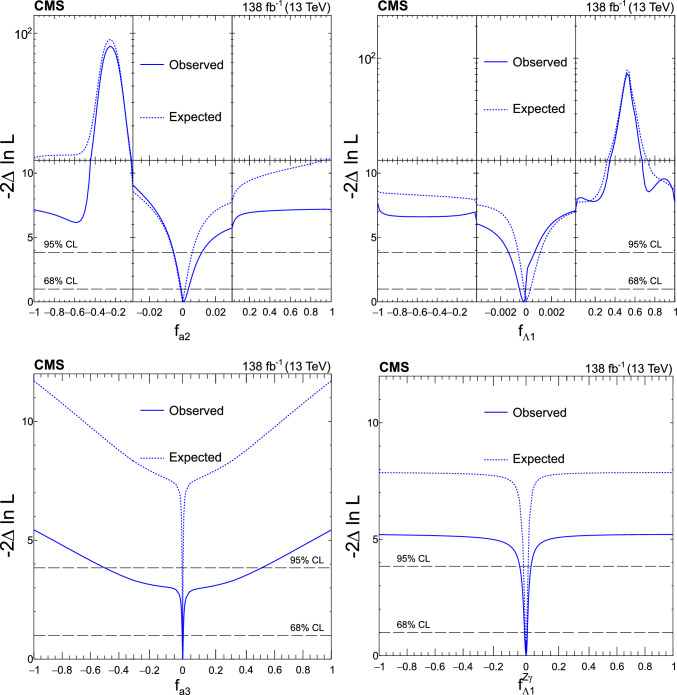
Expected (dashed) and observed (solid) profiled likelihood on $f_{a2}$ (upper left), $f_{\varLambda 1}$ (upper right), $f_{a3}$ (lower left), and $f_{\varLambda 1}^{{\textrm{Z}} {{\upgamma }}}$ (lower right) using Approach 1. In each case, the signal strength modifiers are treated as free parameters. The dashed horizontal lines show the 68 and 95% $\text {CL}$ regions. Axis scales are varied for $f_{a2}$ and $f_{\varLambda 1}$ to improve the visibility of important features

**Fig. 9:**
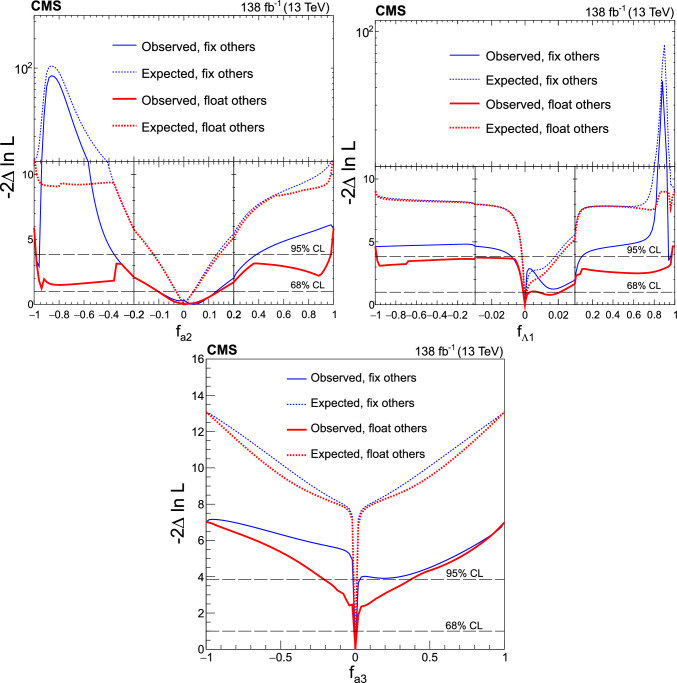
Expected (dashed) and observed (solid) profiled likelihood on $f_{a2}$ (upper left), $f_{\varLambda 1}$ (upper right) and $f_{a3}$ (bottom) using Approach 2. The other two anomalous coupling cross section fractions are either fixed to zero (blue) or left floating in the fit (red). In each case, the signal strength modifiers are treated as free parameters. The dashed horizontal lines show the 68 and 95% $\text {CL}$ regions. Axis scales are varied for $f_{a2}$ and $f_{\varLambda 1}$ to improve the visibility of important features

**Table 7:** Summary of constraints on the anomalous HVV and Hgg coupling parameters with the best fit values and allowed 68 and 95% $\text {CL}$ (in square brackets) intervals. For Approach 1, each $f_{ai}$ is studied independently. For Approach 2, each $f_{ai}$ is shown separately with the other two cross section fractions either fixed to zero or left floating in the fit. In each case, the signal strength modifiers are treated as free parameters

Analysis	$f_{ai}$		Observed $(\times 10^{-3})$	Expected $(\times 10^{-3})$
HVV	$f_{a2}$	Best fit	0.5	0.0
Approach 1		68% $\text {CL}$	[− 0.8, 3.5]	[− 1.4, 1.3]
		95% $\text {CL}$	[− 5.7, 12.0]	[− 5.2, 6.1]
	$f_{a3}$	Best fit	0.9	0.0
		68% $\text {CL}$	[− 2.7, 4.1]	[− 0.7, 0.7]
		95% $\text {CL}$	[− 553.0, 561.0]	[− 2.8, 2.9]
	$f_{\varLambda 1}$	Best fit	− 0.2	0.0
		68% $\text {CL}$	[− 0.5, 0.0]	[− 0.2, 0.5]
		95% $\text {CL}$	[− 1.4, 0.7]	[− 0.6,1.4]
	$f_{\varLambda 1}^{{\textrm{Z}} {{\upgamma }}}$	Best fit	3.0	0.0
		68% $\text {CL}$	[− 11.0, 9.1]	[− 5.0, 3.8]
		95% $\text {CL}$	[− 55.0, 42.0]	[− 14.0, 11.0]
HVV	$f_{a2}$	Best fit	38.0	0.0
Approach 2		68% $\text {CL}$	[− 112.2, 129.3]	[− 30.9, 37.5]
(Fix others)		95% $\text {CL}$	[− 376.6, 430.0]$\cup$[− 989.2, − 826.3]	[− 126.1, 136.8]
	$f_{a3}$	Best fit	0.8	0.0
		68% $\text {CL}$	[− 0.8, 3.5]	[− 0.8, 1.1]
		95% $\text {CL}$	[− 7.6, 58.8]	[− 3.4, 4.3]
	$f_{\varLambda 1}$	Best fit	− 0.15	0.0
		68% $\text {CL}$	[− 1.21, 0.16]	[− 0.4, 0.4]
		95% $\text {CL}$	[− 19.5, 118.5]$\cup$[909.9, 964.1]	[− 1.7, 18.9]
HVV	$f_{a2}$	Best fit	− 1.0	0.0
Approach 2		68% $\text {CL}$	[− 104.1, 139.9]	[− 31.1, 39.8]
(Float others)		95% $\text {CL}$	[− 986.4, 981.2]	[− 127.5, 148.7]
	$f_{a3}$	Best fit	0.34	0.0
		68% $\text {CL}$	[− 0.69, 3.4]	[− 1.0, 1.2]
		95% $\text {CL}$	[− 201.3, 361.5]	[− 4.3, 5.3]
	$f_{\varLambda 1}$	Best fit	− 0.1	0.0
		68% $\text {CL}$	[− 1.08, 3.78]$\cup$[7.2, 20.7]	[− 0.4, 0.9]
		95% $\text {CL}$	[− 994.8, 993.9]	[− 1.9, 21.4]
Hgg	$f_{a3}^{\textrm{ggH}}$	Best fit	− 34	0
		68% $\text {CL}$	[− 721, 383]	[− 1000, 1000]
		95% $\text {CL}$	[− 1000, 1000]	[− 1000, 1000]

**Fig. 10:**
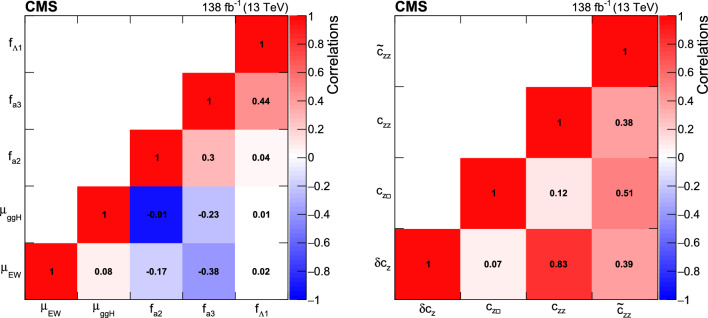
The observed correlation coefficients between HVV anomalous coupling cross section fractions and signal strength modifiers (left) and between SMEFT Higgs basis coupling parameters (right)

**Fig. 11:**
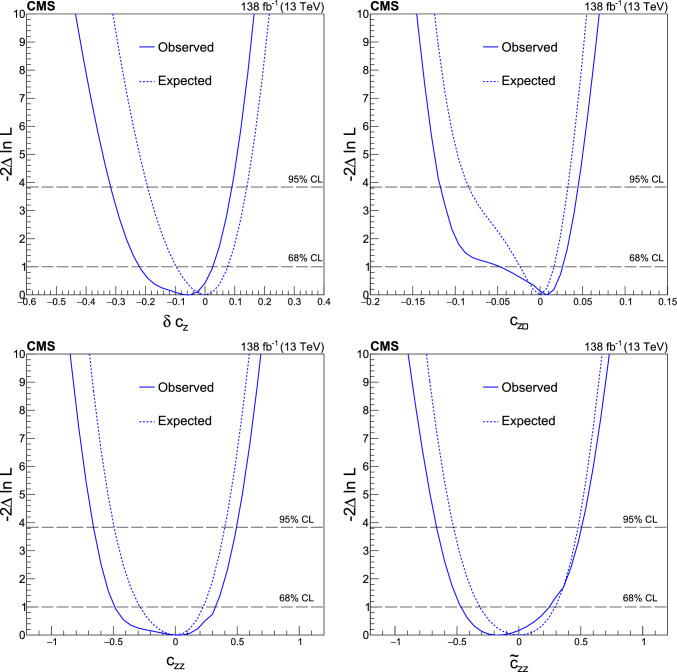
Expected (dashed) and observed (solid) profiled likelihood on the $\delta c_{\text {z}}$ (upper left), $c_{\text {z}\Box }$ (upper right), $c_{\text {zz}}$ (lower left), and $\tilde{c}_{\text {zz}}$ (lower right) couplings of the SMEFT Higgs basis. All four couplings are studied simultaneously. The dashed horizontal lines show the 68 and 95% $\text {CL}$ regions

The optimization and validation of the analysis were performed using simulation and data in CRs. The data in the SRs were examined once all details of the analysis were finalized. For the final results, we perform a binned maximum likelihood fit to the data combining all channels and data-taking periods. The statistical approach was developed by the ATLAS and CMS Collaborations in the context of the LHC Higgs Combination Group [101]. The likelihood function is defined for candidate events as:

22 $$\begin{aligned}{} & {} {\mathcal {L}}(\text {data} | \mu _{\textrm{ggH}}, \mu _{\text {EW}}, f_{ai}, \theta ) \nonumber \\{} & {} \quad =\prod _{\textrm{j}}\text {Poisson}(n_{\textrm{j}}|s_{\textrm{j}}(\mu _{\textrm{ggH}}, \mu _{\text {EW}}, f_{ai}, \theta )+b_{\textrm{j}}(\theta )) p(\tilde{\theta } | \theta ), \nonumber \\ \end{aligned}$$

where j runs over all bins and $n_{\textrm{j}}$ is the observed number of data events in each bin. Total signal and background expectations in each bin are represented by $s_{\textrm{j}}$ and $b_{\textrm{j}},$ respectively. The individual signal and background processes considered in each category are described using binned templates of multidimensional discriminants as described in Sect. 8. Each signal process is parametrized as a linear combination of terms originating from the SM, and anomalous couplings and their interference. The signal expectation depends on the parameters $\mu _{\textrm{ggH}},$ $\mu _{\text {EW}},$ and $f_{ai}$, and is constrained by the data fit. Both the signal and background expectations are functions of $\theta ,$ which represents the full set of nuisance parameters corresponding to the systematic uncertainties. The CRs described in Sect. 6 are included in the fit in the form of single bins, representing the number of events in each CR.

The $\mu _{\textrm{ggH}}$ and $\mu _{\text {EW}}$ parameters correspond to the Higgs boson signal strength modifiers for the ggH and VBF/VH signals, respectively. Signal yields for the VBF and VH processes are related to each other because the same HVV couplings enter both in production and decay of the Higgs boson. The ggH signal is initiated predominantly by the top fermion couplings and is unrelated to the VBF and VH production mechanisms. As the signal strength modifiers are free parameters in the fit, the overall signal event yield is not used to discriminate between alternative signal hypotheses. The $f_{ai}$ parameter corresponds to the anomalous coupling cross section fraction and determines the shape of the signal expectation. The cross section fraction for the SM coupling is simply taken as $1 - \left| {f_{ai}}\right| .$ In Approach 1, the SM and just one anomalous HVV coupling are included, and each $f_{ai}$ is thus studied independently. Depending on the particular anomalous coupling under investigation, $f_{ai}$ may represent $f_{a2}$, $f_{a3}$, $f_{\varLambda 1}$, or $f_{\varLambda 1}^{{\textrm{Z}} {{\upgamma }}}$. For Approach 2, the SM and three anomalous HVV couplings are included. In this case, $f_{ai}$ represents $f_{a2}$, $f_{a3}$ and $f_{\varLambda 1}$, which are studied simultaneously. It is explicitly required that $\left| {f_{a2}}\right| + \left| {f_{a3}}\right| + \left| {f_{\varLambda 1}}\right| \le 1$ to avoid probing an unphysical parameter space. Finally, there is just one anomalous coupling corresponding to $f_{a3}^{\textrm{ggH}}$ to consider for the Hgg vertex. For this study, we also include the effect of the ${\textit{CP}}$-odd HVV anomalous coupling on the VBF process. This is achieved by including $f_{a3}$ as a free parameter in the fit. The $p(\tilde{\theta }|\theta )$ are the probability density functions (PDFs) for the observed values of the nuisance parameters, $\tilde{\theta },$ obtained from calibration measurements. The systematic uncertainties that affect only the normalizations of the signal and background processes are treated as PDFs following a log-normal distribution, whereas shape-altering systematic uncertainties are treated as Gaussian PDFs [101].

Additional interpretations in terms of the SMEFT Higgs and Warsaw basis coupling parameters are also considered using Eqs. (7–10) and Eqs. (11–14), respectively. In each case, four independent couplings are studied simultaneously and the effect of the couplings on the total width of the Higgs boson is taken into account. For the $f_{ai}$ measurements, this effect is absorbed by the signal strength modifiers. A parameterization of the partial widths of the main Higgs boson decay modes as a function of the couplings is used to determine the effect on the Higgs boson width [24, 28].

The likelihood is maximized with respect to the signal modifier parameters and with respect to the nuisance parameters. Confidence level ($\text {CL}$) intervals are determined from profile likelihood scans of the respective parameters. The allowed 68% and 95% $\text {CL}$ intervals are defined using the set of parameter values at which the profile likelihood function $-2\varDelta \ln {{\mathcal {L}}} = 1.00$ and 3.84 [102], respectively, for which exact coverage is expected in the asymptotic limit [103]. The likelihood value at a given $f_{ai}$ is determined by the shape of the signal hypothesis and the relative signal event yields between categories. Expected results are obtained using the Asimov data set [104] constructed using the SM values of the signal modifier parameters.

For Approach 1, where we assume $a_{i}^\textrm{ZZ}=a_{i}^{\textrm{WW}},$ the expected and observed $f_{a2}$, $f_{a3}$, $f_{\varLambda 1}$, and $f_{\varLambda 1}^{{\textrm{Z}} {{\upgamma }}}$ likelihood scans are shown in Fig. 8. Significant interference effects for negative values of $f_{a2}$, around $-0.25,$ and positive values of $f_{\varLambda 1}$, around 0.5, are evident. Relatively large changes in the signal shape with respect to the SM are predicted at these values. Also evident are narrow minima around $f_{ai}$  = 0. The anomalous coupling terms in Eq. (1) have a $q_{i}^{2}$ dependence, which can be larger at the VBF/VH production vertex than at the Higgs decay vertex. This causes the cross section and the shape of the VBF/VH signal hypothesis to change rapidly with $f_{ai}$. For $f_{\varLambda 1}^{{\textrm{Z}} {{\upgamma }}}$, there are no anomalous effects at the Higgs decay vertex and so the only structure present is the narrow minimum related to the VBF/VH production vertex. The axis scales are varied to improve the visibility of important features for $f_{a2}$ and $f_{\varLambda 1}$. For Approach 2, where the SU(2) x U(1) coupling relationships from Eqs. (2–6) are adopted, the expected and observed $f_{a2}$, $f_{a3}$ and $f_{\varLambda 1}$ likelihood scans are shown in Fig. 9. The results are shown for each $f_{ai}$ separately with the other two $f_{ai}$ either fixed to zero or left floating in the fit. The measured values of the signal strength parameters correspond to $\mu _{\text {EW}}=0.9^{+0.19}_{-0.24}$ and $\mu _{\textrm{ggH}}=0.9^{+0.38}_{-0.20}$ when all parameters float simultaneously. It is notable that the observed $-2\varDelta \ln {{\mathcal {L}}}$ profile values are generally lower than expected. This is consistent with a downward statistical fluctuation in the number of VBF and VH events. The lowest $\mu _{\text {EW}}$ value measured is 0.82 for the Approach 1 $f_{a3}$ fit which can be compared with the highest value of 0.97 for the corresponding $f_{\varLambda 1}$ fit. In each case, the uncertainty in $\mu _{\text {EW}}$ is about 20% and as such all fitted values are consistent with both the SM and each other. More generally, all anomalous HVV coupling parameter measurements are consistent with the expectations for the SM Higgs boson. The p-value compatibility of the full Approach 2 fit, where all signal parameters float simultaneously, with the SM is 91%. A summary of constraints on the anomalous HVV coupling parameters with the best fit values and allowed 68% and 95% $\text {CL}$ intervals are shown in Table 7. The most stringent constraints on the HVV anomalous coupling cross section fractions are at the per mille level. Some constraints are less stringent than expected due to the fitted values of $\mu _{\text {EW}}$ being lower than the SM expectation. The observed correlation coefficients between HVV anomalous coupling cross section fractions and signal strength modifiers are displayed in Fig. 10.

**Fig. 12:**
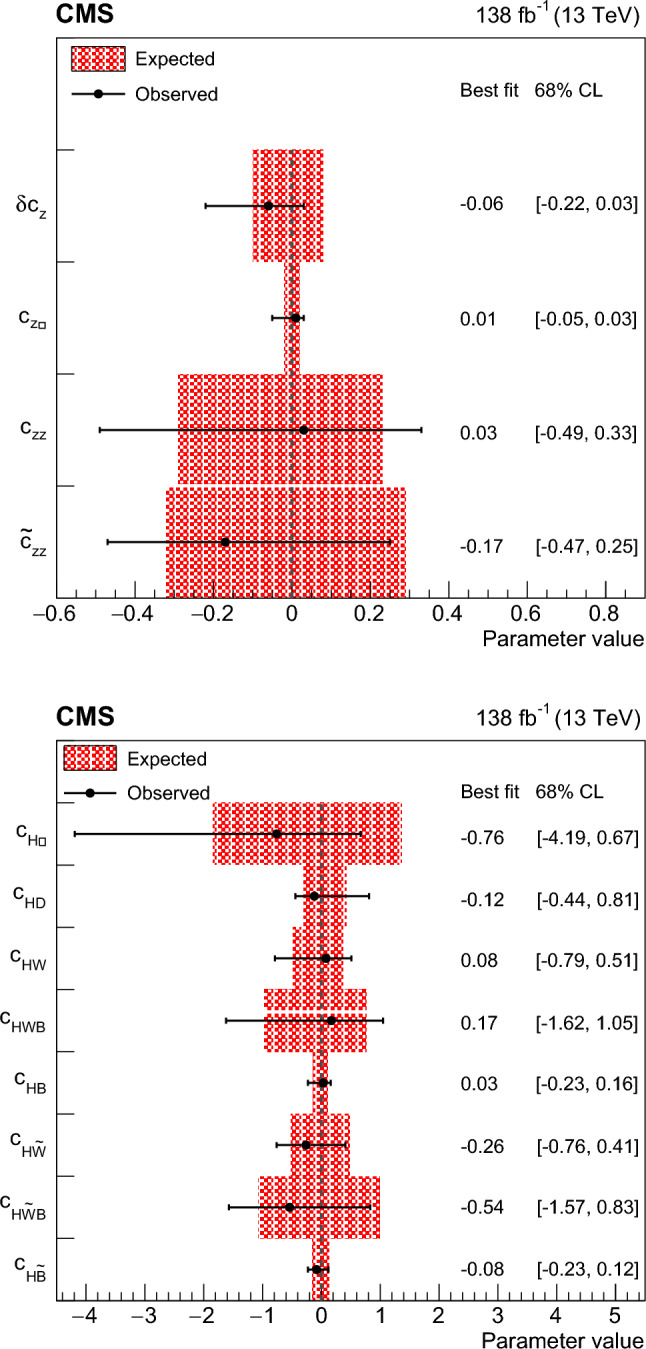
Summary of constraints on the SMEFT Higgs (upper) and Warsaw (lower) basis coupling parameters with the best fit values and 68% $\text {CL}$ uncertainties. For the Warsaw basis, only one of $c_{\text {HW}}$ , $c_{\text {HWB}},$ and $c_{\text {HB}}$ is independent, the same is also true for $c_{\text {H}\tilde{\text {W}}},$ $c_{\text {H}\tilde{\text {W}}\text {B}},$ and $c_{\text {H}\tilde{\text {B}}}$

For the SMEFT Higgs basis interpretation, the expected and observed constraints on the $\delta c_{\text {z}},$ $c_{\text {z}\Box },$ $c_{\text {zz}},$ and $\tilde{c}_{\text {zz}}$ coupling parameters are shown in Fig. 11. Table 8 presents a summary of the constraints on the couplings whereas Fig. 10 reports the observed correlation coefficients between them. For the Warsaw basis interpretation, the expected and observed constraints on the $c_{\text {H}\Box },$ $c_{\text {HD}},$ $c_{\text {HW}},$ $c_{\text {HWB}},$ $c_{\text {HB}},$ $c_{\text {H}\tilde{\text {W}}},$ $c_{\text {H}\tilde{\text {W}}\text {B}},$ and $c_{\text {H}\tilde{\text {B}}}$ coupling parameters are presented in Table 9. To cover all the Warsaw basis coupling parameters, three independent fits to the data were performed with a different choice of four independent couplings in each. A summary of the constraints on the SMEFT Higgs and Warsaw basis coupling parameters is presented in Fig. 12.

**Table 8:** Summary of constraints on the SMEFT Higgs basis coupling parameters with the best fit values and 68% $\text {CL}$ uncertainties. All four couplings are studied simultaneously

Coupling	Observed	Expected
$\delta c_{\text {z}}$	$-0.06^{+0.09}_{-0.16}$	$0.00^{+0.08}_{-0.10}$
$c_{\text {z}\Box }$	$0.01^{+0.02}_{-0.06}$	$0.00^{+0.02}_{-0.02}$
$c_{\text {zz}}$	$0.03^{+0.30}_{-0.52}$	$0.00^{+0.23}_{-0.29}$
$\tilde{c}_{\text {zz}}$	$-0.17^{+0.42}_{-0.30}$	$0.00^{+0.29}_{-0.32}$

**Fig. 13:**
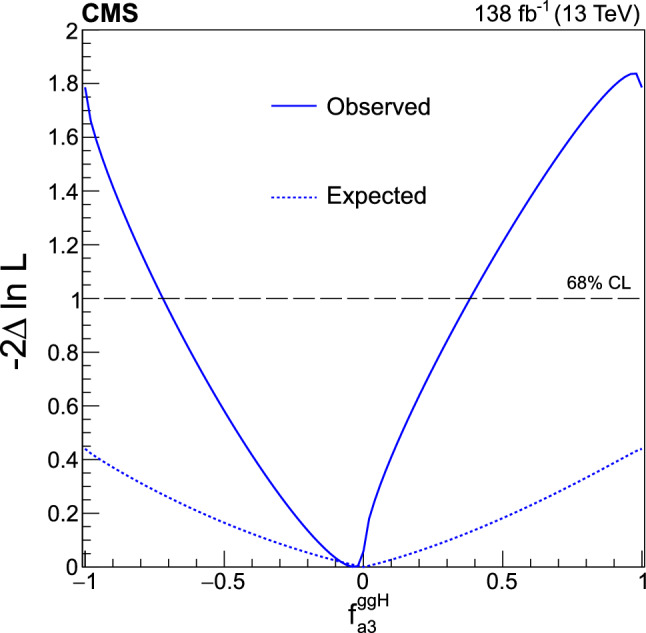
Expected (dashed) and observed (solid) profiled likelihood on $f_{a3}^{\textrm{ggH}}$. The signal strength modifiers and the *CP*-odd HVV anomalous coupling cross section fraction are treated as free parameters. The crossing of the observed likelihood with the dashed horizontal line shows the observed 68% $\text {CL}$ region

Finally, the expected and observed $f_{a3}^{\textrm{ggH}}$ likelihood scans are shown in Fig. 13. The result is consistent with the expectation for a SM Higgs boson. Excluding the effect of the ${\textit{CP}}$-odd HVV anomalous coupling, by fixing $f_{a3}$ to zero, has a negligible effect. For $\left| {f_{a3}^{\textrm{ggH}}}\right|$ approaching unity, the observed $-2\varDelta \ln {{\mathcal {L}}}$ profile values are larger than expected. This is consistent with downward statistical fluctuations in the data for a couple of bins where sensitivity to the $a_{3}$ Hgg coupling contribution is enhanced (Fig. 7 upper). The constraint on the anomalous Hgg coupling parameter with the best fit value and allowed 68% $\text {CL}$ interval is shown in Table 7.

**Table 9:** Summary of constraints on the SMEFT Warsaw basis coupling parameters with the best fit values and 68% $\text {CL}$ uncertainties. Only one of $c_{\text {HW}},$ $c_{\text {HWB}},$ and $c_{\text {HB}}$ is independent, the same is also true for $c_{\text {H}\tilde{\text {W}}},$ $c_{\text {H}\tilde{\text {W}}\text {B}},$ and $c_{\text {H}\tilde{\text {B}}}.$ Three independent fits to the data were performed with a different choice of four independent couplings in each

Coupling	Observed	Expected
$c_{\text {H}\Box }$	$-0.76^{+1.43}_{-3.43}$	$0.00^{+1.37}_{-1.84}$
$c_{\text {HD}}$	$-0.12^{+0.93}_{-0.32}$	$0.00^{+0.43}_{-0.30}$
$c_{\text {HW}}$	$0.08^{+0.43}_{-0.87}$	$0.00^{+0.37}_{-0.48}$
$c_{\text {HWB}}$	$0.17^{+0.88}_{-1.79}$	$0.00^{+0.77}_{-0.96}$
$c_{\text {HB}}$	$0.03^{+0.13}_{-0.26}$	$0.00^{+0.11}_{-0.14}$
$c_{\text {H}\tilde{\text {W}}}$	$-0.26^{+0.67}_{-0.50}$	$0.00^{+0.48}_{-0.52}$
$c_{\text {H}\tilde{\text {W}}\text {B}}$	$-0.54^{+1.37}_{-1.03}$	$0.00^{+0.99}_{-1.07}$
$c_{\text {H}\tilde{\text {B}}}$	$-0.08^{+0.20}_{-0.15}$	$0.00^{+0.15}_{-0.16}$

## Summary

This paper presents a study of the anomalous couplings of the Higgs boson (H) with vector bosons, including ${\textit{CP}}$ violating effects, using its associated production with hadronic jets in gluon fusion, electroweak vector boson fusion, and associated production with a W or Z boson, and its subsequent decay to a pair of W bosons. The results are based on the proton–proton collision data set collected by the CMS detector at the LHC during 2016–2018, corresponding to an integrated luminosity of 138$\,\text {fb}^{-1}$ at a center-of-mass energy of 13TeV. The analysis targets the different-flavor dilepton $({\textrm{e}} {{\upmu }})$ final state, with kinematic information from associated jets combined using matrix element techniques to increase sensitivity to anomalous effects at the production vertex. Dedicated Monte Carlo simulation and matrix element reweighting provide modeling of all kinematic features in the production and decay of the Higgs boson with full simulation of detector effects. A simultaneous measurement of four Higgs boson

couplings to electroweak vector bosons has been performed in the framework of a standard model effective field theory. All measurements are consistent with the expectations for the standard model Higgs boson and constraints are set on the fractional contribution of the anomalous couplings to the Higgs boson cross section. The most stringent constraints on the HVV anomalous coupling cross section fractions are at the per mille level. These results are in agreement with those obtained in the $\textrm{H}\rightarrow \textrm{ZZ}$ and $H\rightarrow \uptau \uptau$ channels, and also significantly surpass those of the previous $\textrm{H}\rightarrow \textrm{WW}$ anomalous coupling analysis from the CMS experiment in both scope and precision.

## Data Availability

Data cannot be made available for reasons disclosed in the data availability statement. [Author’s comment: Release and preservation of data used by the CMS Collaboration as the basis for publications is guided by the CMS policy as stated in the “CMS data preservation, re-use and open access policy.]

## Code Availability Statement

This manuscript has no associated code/software. [Author’s comment: There is no code availability statement.]
